# Plasmid-Encoded Tetracycline Efflux Pump Protein Alters Bacterial Stress Responses and Ecological Fitness of *Acinetobacter oleivorans*

**DOI:** 10.1371/journal.pone.0107716

**Published:** 2014-09-17

**Authors:** Hyerim Hong, Jaejoon Jung, Woojun Park

**Affiliations:** Department of Environmental Science and Ecological Engineering, Korea University, Seoul, Republic of Korea; University of Illinois at Chicago College of Medicine, United States of America

## Abstract

Acquisition of the extracellular tetracycline (TC) resistance plasmid pAST2 affected host gene expression and phenotype in the oil-degrading soil bacterium, *Acinetobacter oleivorans* DR1. Whole-transcriptome profiling of DR1 cells harboring pAST2 revealed that all the plasmid genes were highly expressed under TC conditions, and the expression levels of many host chromosomal genes were modulated by the presence of pAST2. The host energy burden imposed by replication of pAST2 led to (i) lowered ATP concentrations, (ii) downregulated expression of many genes involved in cellular growth, and (iii) reduced growth rate. Interestingly, some phenotypes were restored by deleting the plasmid-encoded efflux pump gene *tetH,* suggesting that the membrane integrity changes resulting from the incorporation of efflux pump proteins also resulted in altered host response under the tested conditions. Alteration of membrane integrity by *tetH* deletion was shown by measuring permeability of fluorescent probe and membrane hydrophobicity. The presence of the plasmid conferred peroxide and superoxide resistance to cells, but only peroxide resistance was diminished by *tetH* gene deletion, suggesting that the plasmid-encoded membrane-bound efflux pump protein provided peroxide resistance. The downregulation of fimbriae-related genes presumably led to reduced swimming motility, but this phenotype was recovered by *tetH* gene deletion. Our data suggest that not only the plasmid replication burden, but also its encoded efflux pump protein altered host chromosomal gene expression and phenotype, which also alters the ecological fitness of the host in the environment.

## Introduction

Plasmids carrying antibiotic resistance genes are one of the primary sources of multidrug resistance in pathogens [1], [2]. Plasmids can be transferred to microorganisms through horizontal gene transfer (HGT), by methods such as conjugation and transformation, which can occur inside the hosts, as well as in the environment [3]–[5]. Many conjugative plasmids harboring antibiotic resistance genes have been shown to be present in wastewater treatment plants (WWTP) [6]–[10]. Antibiotics, including tetracycline (TC), could exert selective pressure on these plasmids and facilitate the spread of antibiotic resistance genes in the environment [11]–[13]. TC prevents bacterial growth by binding to a single site on the 30S ribosomal subunit, and preventing attachment of aminoacyl tRNA molecules to the ribosome [14]. TC-related antibiotics have been applied in clinics, and in agricultural and aquatic settings for disease control and animal growth promotion, and are frequently detected in the effluent from WWTPs [15]–[17]. TC resistance could be acquired by three known mechanisms: energy-driven efflux pumps, ribosomal protection proteins, and TC-modifying enzymes [18]. Efflux pumps encoded by several genes, such as *tetA, tetC, tetE, tetG,* and *tetH,* have been reported to be a major mechanism of TC resistance; these genes are frequently located in plasmids and transposons [19]–[21].

Antibiotic resistance is known to exert a metabolic cost on bacteria, and antibiotic-resistant cells often show a reduced growth rate [22], [23]. The correlation between fitness cost and antibiotic resistance has been extensively reviewed [24]–[26]. Previously, we demonstrated that not only bacterial growth, but also many cellular processes including quorum sensing, motility, and stress response could be affected by acquiring antibiotic resistance [27], [28]. A newly described tetracycline resistance plasmid, pAST2, was isolated from an activated sludge, and its entire plasmid genome was characterized [29]. The pAST2 plasmid encodes the TC efflux pump, *tetH*, and *tetR* genes that confer tetracycline resistance to the host. Expression of the *tetH* gene is known to be regulated by a repressor, TetR [30], [31]. Based on our bioinformatics study, this *tetH-tetR* module originated in bovine and swine pathogens such as *Pasteurella multocida*, *Mannheimia haemolytica*, and *Actinobacillus pleuropneumoniae* [32], [33]. Recently, we demonstrated that the acquisition of this plasmid alters the phenotypic characteristics of the oil-degrading microbe *Acinetobacter oleivorans* DR1 [29]. Presence of the plasmid incurred high ecological costs for phenotypic and physiological functions in *A. oleivorans* DR1. This observation is consistent with the fact that antibiotic resistance plasmids alter the expression of host chromosomal genes and ecological adaptation in *Salmonella* [34].

The aims of this study were (i) to gain insight into the link between changes in host chromosomal expression and phenotypic changes in the presence of the pAST2 plasmid, and (ii) to distinguish the metabolic costs incurred by the plasmid replication burden and by changes in membrane integrity caused by addition of the plasmid-encoded tetracycline efflux pump. We compared four RNA-Seq transcriptomes of the wild-type and plasmid-harboring cells in the presence and absence of TC. To understand the fitness costs of the efflux pump, a *tetH-tetR* knockout plasmid was generated and tested under different environmental conditions. Our findings provide evidence that the expression of many host chromosomal genes can be modulated by a foreign TC resistance plasmid, altering the host’s biological fitness. We also confirm that the plasmid-encoded efflux pump protein alone can influence host phenotype by affecting membrane integrity and permeability.

## Results

### 1. RNA-Seq-based transcriptional profiling of *A. oleivorans* DR1 harboring pAST2

To identify altered expression of genes on the host chromosome in the presence of the plasmid, we conducted RNA-Seq whole-transcriptome analysis using DR1 cells harboring pAST2 in the presence and absence of TC. TC was applied to each exponentially grown cell at a minimal inhibitory concentration (MIC, 1 µg/ml) for 15 min. Fifteen min was determined to be an optimal incubation time so as not to cause serious cell-death by tetracycline in liquid culture. RNA-Seq analyses were performed with wild type DR1, and wild type carrying pAST2, TC-treated DR1, and TC-treated DR1 (pAST2). They were designated as DR1, DR1 (pAST2), DR1-TC, and DR1 (pAST2)-TC, respectively. The total number of reads from DR1, DR1 (pAST2), DR1-TC, and DR1 (pAST2)-TC was 6583302, 36127802, 17509940, and 20507109, respectively. The sequencing coverage for each sample was 57, 868, 151, and 177-fold, respectively. More than 99% of the reads were mapped to the reference genome sequence, which indicates satisfactory sequencing quality. Overall gene expression profiles indicated that dramatic down-regulation occurred in the presence of pAST2 (Figure S1A). With the plasmid, a total of 275 genes were upregulated, and 2125 genes were downregulated (Table 1). However, the majority of genes were upregulated in DR1 cells with pAST2 in the presence of TC (Figure S1B, S1C). This level of upregulation was not observed in the absence of the plasmid (Figure S1D, Table 1).

**Table 1:** RNA-Seq-based transcriptional profiling.

	Fold change			
	DR1(pAST2)/DR1	DR1(pAST2)-TC/DR1-TC	DR1(pAST2)-TC/DR1(pAST2)	DR1-TC/DR1
**Total upregulated genes**	275	1222	2492	713
**Total downregulated genes**	2125	284	115	881
**COG category containing the highest** **proportion of upregulated genes**	–	F	K	N
**COG category containing the highest** **proportion of downregulated genes**	K, T	A, J	N	D, F, H, M

DR1 cells harboring pAST2 showed that transcription-related (COG K) and signal transduction (COG T) genes were downregulated (Figure S2A, Table 1). In contrast, the presence of the plasmid under TC conditions increased expression of many genes in categories F (nucleotide metabolism and transport) and K (transcription), whereas a number of genes in categories A (RNA processing and modification), J (translation), and N (motility-related) were downregulated (Figure S2B, S2C). The wild-type strain under TC repressed the expression of cell cycle control (COG D), nucleotide metabolism and transport (COG F), cell wall structure and outer membrane (COG M), and coenzyme metabolism (COG H) gene expression (Figure S2D). Our data show that the presence of the plasmid could modulate host chromosomal expression.

All the genes on the plasmid appeared to be actively upregulated by more than 2 fold under TC conditions (Table 2). Consistent with our previous observation that the *tetH* promoter was strongly induced at sub-MIC concentrations (0.5 µg/ml) of TC [29], the *tetH* gene was the most strongly upregulated gene (35 fold). Interestingly, mobilization protein A showed the second highest increase in expression (29 fold) under TC conditions, which suggested that TC could facilitate plasmid transfer in the environment.

**Table 2:** Gene expression (fold change) values of pAST2 under tetracycline treatment conditions in *A. oleivorans* DR1.

Gene/orf	Position	Product	RPKMFold change	
			DR1(pAST2)-TC	DR1(pAST2)	pAST2-TC/pAST2
*repB*	71–1063	plasmid replication protein B	493.66	127.81	3.86
*tnp*	1185–2069	transposase	1470.09	768.56	1.91
*tetH*	2410–3612	major facilitator transporter	431.35	12.18	35.41
*tetR*	3704–4327	tetracycline repressor protein	814.01	161.14	5.05
*orf1*	4330–5274	hypothetical protein	465.42	21.75	21.40
*orf2*	5274–5876	hypothetical protein	426.90	15.44	27.65
*orf3*	6071–6595	conserved hypothetical protein	326.26	44.77	7.29
*orf4*	6670–7065	predicted protein	346.53	39.33	8.81
*orf5*	7040–7918	predicted protein	299.74	43.93	6.82
*cueR/merR*	8005–8370	Cu(I)-responsive transcriptional regulator	273.41	42.31	6.46
*orf6*	8631–9443	hypothetical protein	502.21	71.42	7.03
*orf7*	9535–9864	transcriptional regulator, XRE family	601.36	163.10	3.69
*orf8*	9855–10184	helix-turn-helix domain protein	330.11	43.01	7.68
*mobA*	10385–10543	mobilization protein A	151.37	5.22	29.00
*mobC*	10533–10898	mobilization protein C	285.72	18.38	15.55
*orf9*	11231–11818	hypothetical protein	305.42	47.02	6.50

### 2. Plasmid-mediated tetracycline resistance consumes intracellular energy in bacterial cells

**Figure 1:**
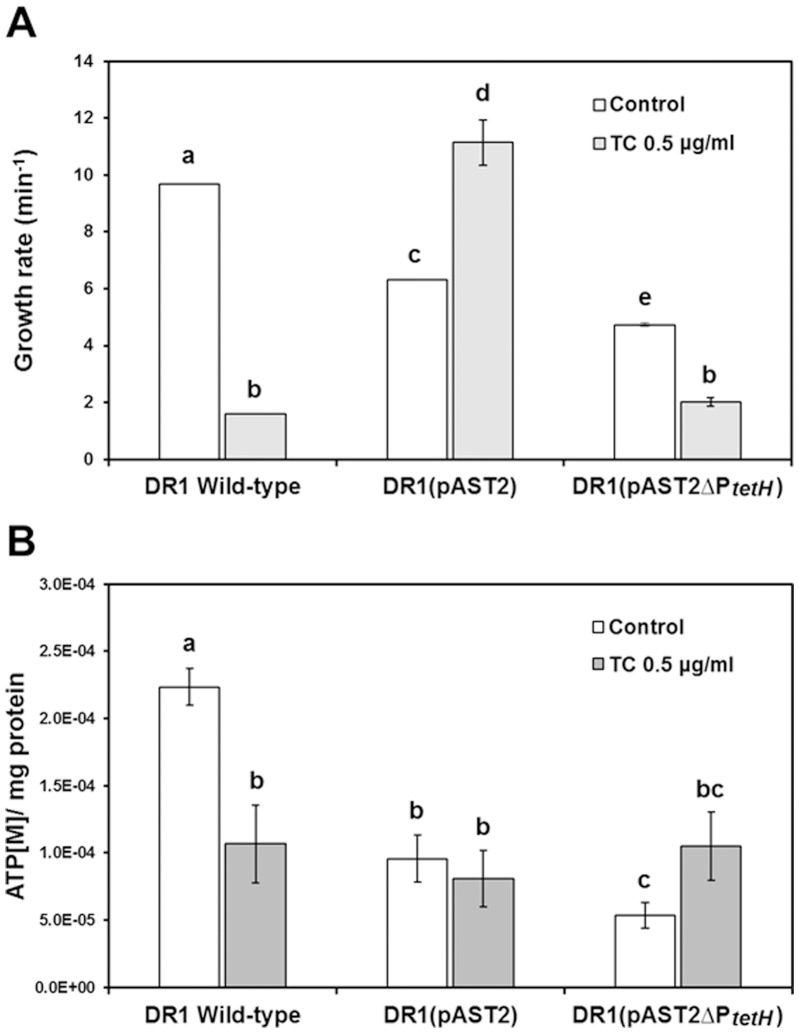
Determination of bacterial growth rates and ATP concentrations in DR1 strains. (A) Growth of DR1 cells in nutrient media containing tetracycline. Cells were grown at 30°C in nutrient media supplemented with or without TC (sub-MIC concentration). The growth rate of each strain was monitored by measuring the optical density (OD) of cultures at 600 nm. Error bars indicate the standard deviation of three independent experiments. (B) Influence of plasmid on ATP generation. The ATP concentration is expressed as molar concentration per mg of protein. All data show the average of three replicates, and one standard deviation is shown. Statistical analyses were conducted using Student’s *t*-test. A letter on the bar graph indicates the level of significance. Bars denoted by the same letter are not statistically significant (*p*>0.05).

Because our transcriptomic data showed that many RNA polymerase-related genes were significantly downregulated in DR1 (pAST2), we hypothesized that presence of the plasmid imposed an energy burden on the host (Table 3). However, addition of TC reversed this tendency, indicating that cells harboring the plasmid actively transcribed many defense genes under TC condition. ATP-related genes were also down-regulated in the presence of the plasmid, which could not be seen under TC (Table 3). Reduction of ATP concentration could be expected with pAST2. However, no clear pattern was observed in the presence of TC. To test this, growth rate and intracellular ATP concentrations were measured with or without TC (Figure 1). Growth rate was significantly reduced in the presence of the plasmid regardless of the presence of the *tetH* gene, which suggested that the replication burden was the major cause of the growth defect (Figure 1A). However, the degree of growth defect differed in the presence and absence of the *tetH* gene, which indicated that expression and membrane incorporation of the plasmid-encoded efflux pump imposed a metabolic cost on the host. As expected, the wild-type strain with pAST2 gained a growth advantage under TC conditions and deletion of the *tetH* gene from the plasmid decreased growth under TC conditions (Figure 1A). Accordingly, wild-type DR1 cells had lower ATP levels in the presence of TC (Figure 1B). ATP concentrations were significantly lower with the plasmid regardless of TC presence. It is interesting to note that under TC conditions, plasmid-carrying cells appeared to use energy efficiently for boosting their growth rate, because their growth rate was very high in spite of low ATP levels (Figure 1A, 1B). A further reduction in ATP levels was measured when the *tetH* gene was deleted, compared to plasmid-carrying cells (Figure 1B). Consistent with the growth rate measurements, the addition of the efflux pump appeared to interfere with cellular processes, resulting in lower ATP levels and decreased growth.

**Table 3:** Gene expression (fold change) values of genes related to transcription, ATPase, ATP synthase, and hexadecane degradation.

Locus_tag	Gene	Product	Fold change	
			DR1(pAST2)/DR1	DR1(pAST2)-TC/DR1-TC
**Transcription-related genes**				
AOLE_16340	*rpoN*	RNA polymerase factor sigma-54	−7.19	4.57
AOLE_04330	*rpoD*	RNA polymerase factor sigma-70	−6.63	1.68
AOLE_12520	–	DNA-directed RNA polymerase	−5.25	1.24
AOLE_12655	*rpoE*	RNA polymerase sigma-24 subunit	−3.04	1.64
AOLE_05735	*rpoH*	RNA polymerase factor sigma-32	−3.00	1.28
AOLE_08705	*rocR*	sigma54 specific transcriptional regulator	−2.00	2.00
**ATPase and ATP synthase-related genes**				
AOLE_17825	*recN*	ATPase	−9.21	1.91
AOLE_18605	*atpI*	ATP synthase I chain family protein	−5.33	−2.75
AOLE_15995	–	putative ATPase	−4.80	1.87
AOLE_08275	*clpS*	ATP-dependent Clp protease adaptor protein	−2.51	1.07
AOLE_18600	*atpB*	F0F1 ATP synthase subunit A	−1.70	1.09
**Hexadecane degradation-related genes**				
AOLE_06655	*putA*	NAD-dependent aldehyde dehydrogenase	−2.79	−7.41
AOLE_10550	*alkB*	alkane 1-monooxygenase	−1.58	2.41
AOLE_14590	*nirB*	3-phenylpropionate dioxygenase ferredoxin	−1.46	1.30
AOLE_03630	*eutG*	alcohol dehydrogenase	1.37	1.77
AOLE_09790	*adhC*	Zn-dependent alcohol dehydrogenase	1.60	4.11
AOLE_14370	*nirB*	rubredoxin-NAD(+) reductase	1.80	2.00

### 3. Loss of hexadecane degradation ability in strain DR1 harboring pAST2

**Figure 2:**
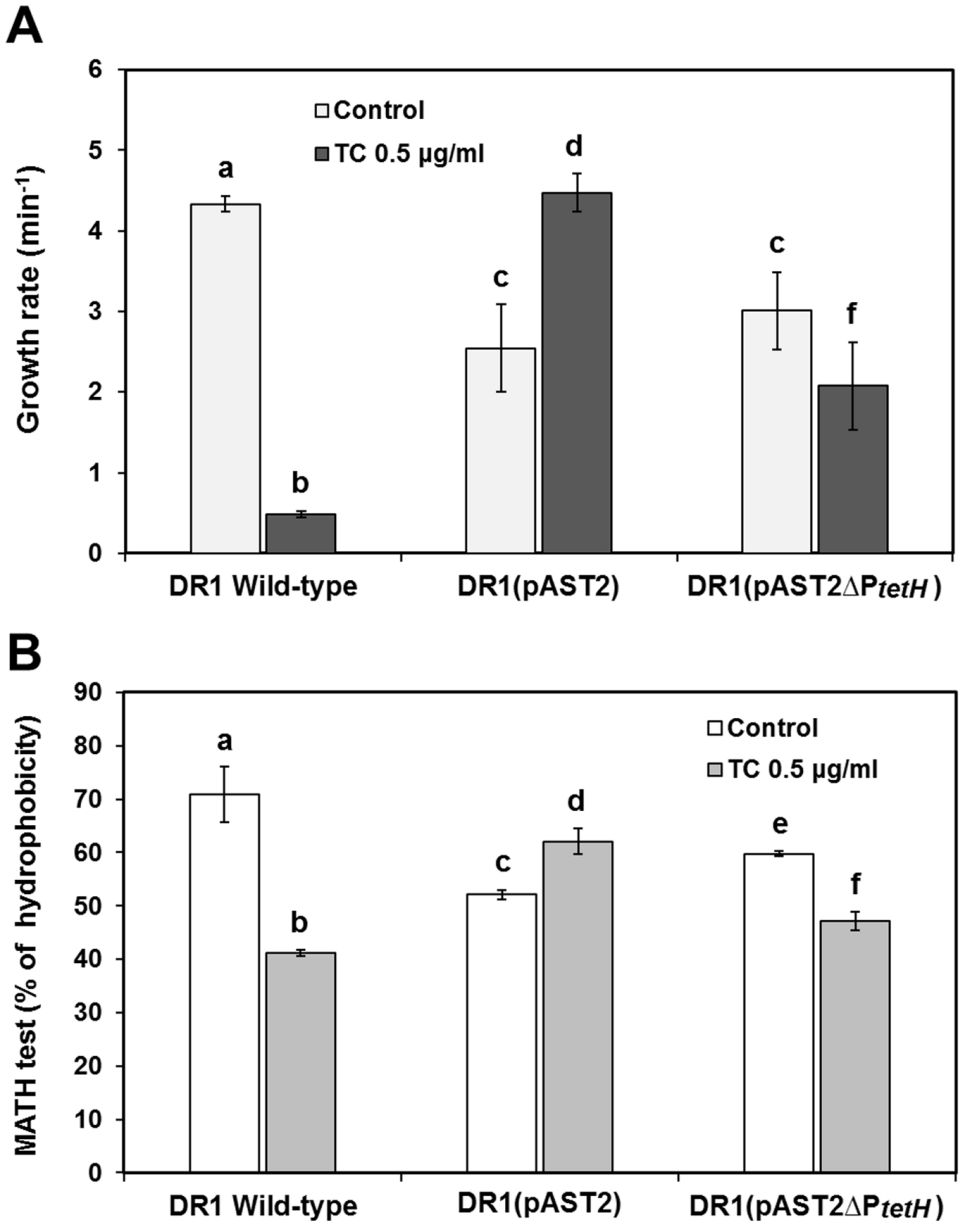
Hexadecane degradation and microbial adherence to n-hexadecane (MATH). (A) Hexadecane degradation rate (min^−1^). Utilization of hexadecane was monitored by measuring the OD_600_. Error bars indicate the standard deviation of three independent experiments. Statistical significance of differences was calculated using Student’s *t*-test. (B) Microbial adhesion to hydrocarbon (MATH) assay. All samples were analyzed in triplicate. Statistical analyses were conducted using Student’s *t*-test. A letter on the bar graph indicates the level of significance. Bars denoted by the same letter are not statistically significant (*p*>0.05).

*Acinetobacter* is well-known for its ability to degrade alkanes [35]–[37]. It has been shown that the hexadecane degradation pathway is present in the *A. oleivorans* DR1 genome [38]–[41]. Many genes involved in hexadecane utilization, including alkane 1-monooxygenase encoded by *alkB* (AOLE_10550) and aldehyde dehydrogenase encoded by *putA* (AOLE_06655) were downregulated in the presence of the plasmid (Table 3). However, under TC conditions, *alkB* gene and Zn-dependent aldehyde dehydrogenase (*adhC*) were upregulated, indicating that different alkane degradation pathways might be used under different conditions. With hexadecane as the sole carbon source and an absence of TC, the growth rates of all he pAST2-harboring cells were lower than those of the wild-type cells (Figure 2A). Both DR1 (pAST2) and DR1 (pAST2ΔP*_tetH_*) showed better utilization of hexadecane than wild-type cells in the presence of TC. However, deletion of the *tetH* gene did not affect growth rate on hexadecane, which suggests that alteration of membrane integrity by TetH is not an important factor for hexadecane utilization. A microbial adhesion to hydrocarbon (MATH) assay was conducted to determine membrane hydrophobicity. MATH values were lower in the presence of pAST2. Thus, deletion of the *tetH* gene altered membrane hydrophobicity (Figure 2B).

### 4. Antibiotic sensitivity and response to oxidative stress in DR1 strains harboring pAST2

Our RNA-Seq analysis showed that many multidrug-efflux pump-related genes were downregulated in the absence of TC (Table S1). Expression of many chromosomal efflux pump genes was repressed in the presence of pAST2. Aminoglycoside resistance efflux pump (AOLE_02025), and two beta-lactamase genes (AOLE_05220 and AOLE_11070) were downregulated 11.9, 11,7 and 7.0 fold, respectively. The pAST2 plasmid appears to elevate expression level of many chromosomal genes involved in stress resistance under TC-amended condition (Table S1). Various chemical compounds were applied to the DR1 wild-type strains and plasmid-harboring strains to determine the effects of the exogenous plasmid on host response to various stresses. Antibiotics were amended at sub-MIC levels. Interestingly, the DR1 strain harboring the plasmid became sensitive to ampicillin in the absence of TC (Figure S3A). This sensitivity was lost when the *tetH* gene was deleted, which showed that TetH is critical for ampicillin sensitivity. Kanamycin gene cassette replacing the *tetH* gene in the plasmid was responsible for kanamycin resistance. Under TC conditions (Figure S3B), the DR1 strain harboring the plasmid appeared to have greater resistance to several antibiotics, with the exception of ampicillin. Cell-wall inhibition by ampicillin thus appears to be strongly affected by the presence of the TetH efflux pump. Our data showed that the plasmid replication burden altered genetic and phenotypic responses of the host to antibiotics; the *tetH-*encoded efflux pump alone could affect these host responses.

**Figure 3:**
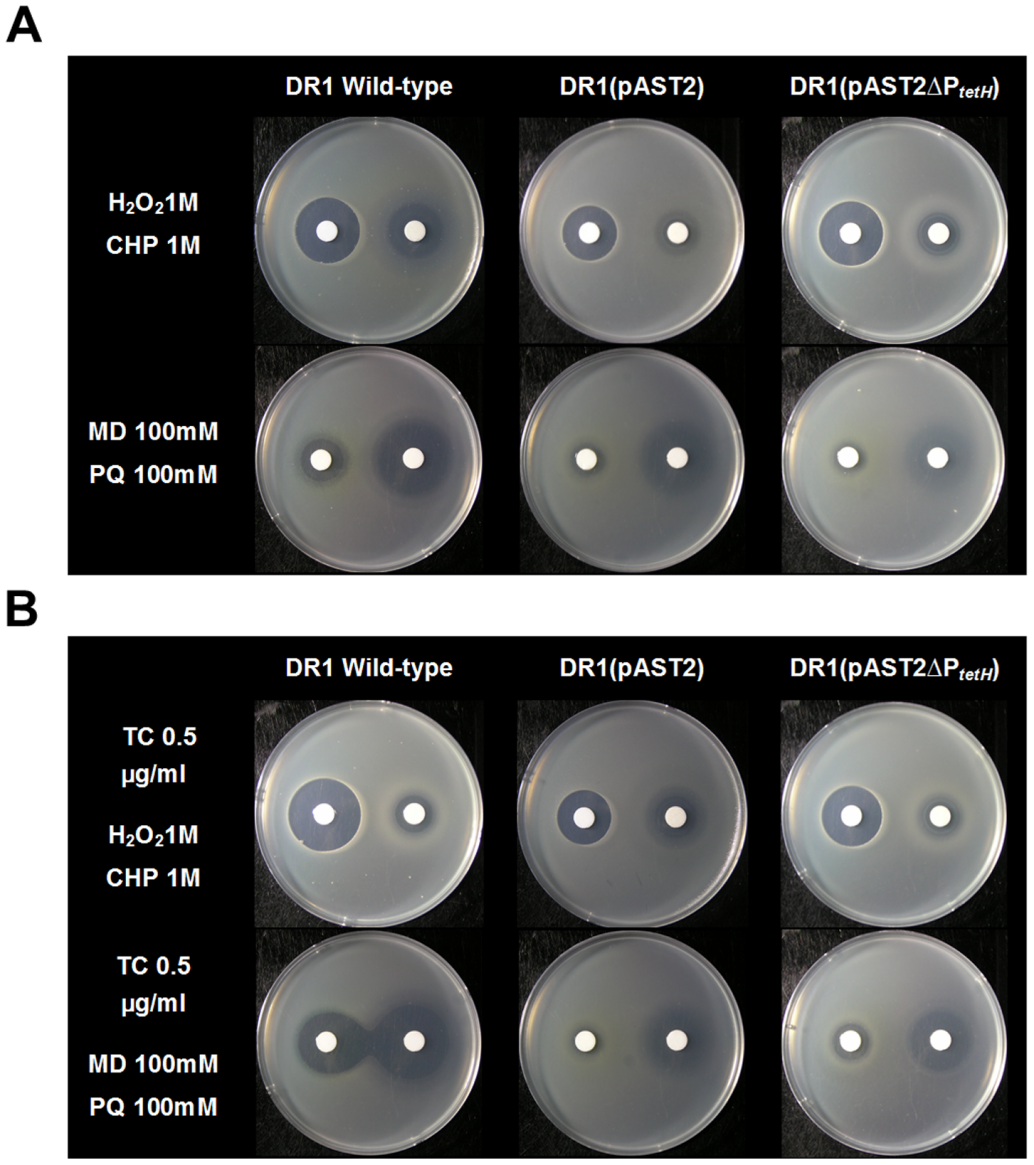
Sensitivity of *Acinetobacter* strains to oxidative stress. (A) Oxidative stress inhibition zone assay in nutrient media (control condition). (B) Oxidative stress inhibition zone assay with TC. Oxidative stress compounds, including superoxide (menadione, MD; paraquat, PQ) and hydrogen peroxide (hydroperoxide, H_2_O_2_; cumene hydroperoxide, CHP), were tested on exponentially grown cells.

Several genes functioning in oxidative stress defense were upregulated in the presence of the plasmid (thioredoxin, 3.6 fold; glutaredoxin, 2.6 fold; catalase, *katG*, 2.4 fold; catalase, *katE,* 2.0 fold; rubredoxin reductase, *nirB*, 1.8 fold) although some oxidative stress-related genes were downregulated (Table S1). The oxidative stress responses of the host harboring the plasmid were tested using compounds such as superoxide [menadione (MD), and paraquat (PQ)] and hydrogen peroxide [hydroperoxide (H_2_O_2_), cumene hydroperoxide (CHP)]. The results showed that DR1 harboring pAST2 had greater resistance to all oxidative stress compounds than wild-type cells and *tetH-*deleted strains (Figure 3A and 3B). DR1 strains, except cells harboring pAST2, were more sensitive to peroxide compounds, which increased the size of the inhibition zone (Figure 3A). Under TC conditions, plasmid-harboring DR1 strains displayed a consistent radius with control, and the wild-type strain was more sensitive to oxidative stress compounds (Figure 3B). Our data demonstrated that the pAST2 plasmid can increase the expression of many stress response-associated genes, reducing sensitivity to oxidative and antibiotic stresses. Moreover, our data provide evidence that the plasmid-mediated efflux pump protein conferred peroxide resistance.

### 5. Additional phenotypic changes caused by the plasmid-encoded TetH efflux pump - motility, membrane permeability, and morphology of fimbria appendages

**Figure 4:**
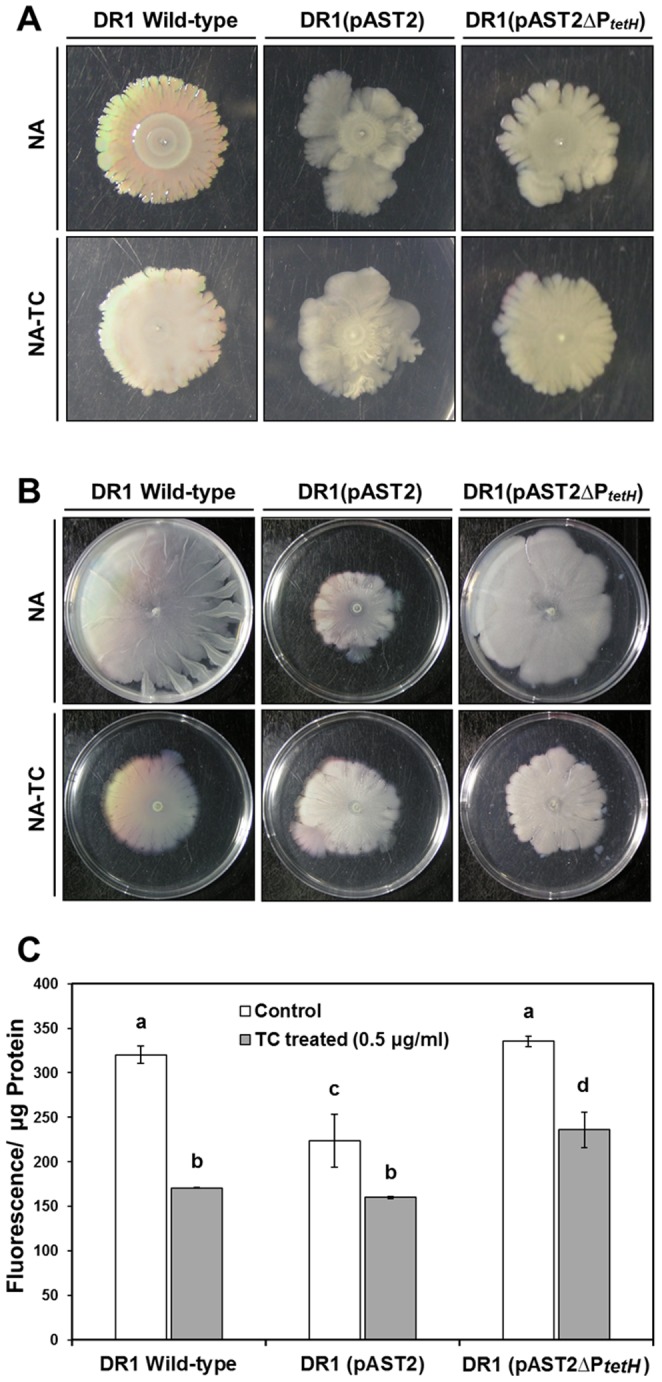
Bacterial capsule-related phenotype test. The plasmid-encoded membrane bound TC efflux pump alters bacterial outer membrane and capsule related appendages. (A) Swarming motility (0.5%), (B) Swimming motility (0.2%), (C) Membrane permeability assay. All quantitative data were obtained in triplicate. A letter on the bar graph indicates the level of significance. Bars denoted by the same letter are not statistically significant (*p*>0.05).

RNA-Seq data showed that expression of many outer membrane proteins (Table S2) and fimbriae/pili-related genes was altered (Table 4). Decreased expression of fimbrial proteins encoded by *fim* genes was consistent with poor motility and decreased surface attachment. [*fimA* (AOLE_09780), *fimC* (AOLE_06715), and *fimT* (AOLE_03010) were downregulated by 7.0, 5.7, and 4.8 fold, respectively (Table 4)]. Swarming motility of wild-type cells showed a distinct branched structure with serrated edges on semi-solid nutrient agar (Figure 4A). However, swarming motility of strain DR1 (pAST2) exhibited a mucoid phenotype with irregular form. Interestingly, the *tetH* mutant strain maintained a branched form similar to the wild-type strain. Under TC conditions, the spreading zones of the three DR1 strains showed highly mucoid phenotypes with exopolysaccharide (EPS) overproduction. Interestingly, cells harboring pAST2 appeared to have reduced swimming motility, but the *tetH* mutant strain showed recovered swimming motility (Figure 4B). On TC-containing swimming agar plates, motility of the *tetH* mutant and wild-type strains was equally reduced.

**Table 4:** Expression profile of bacterial fimbriae-related genes.

Locus_tag	Gene	Product	Fold change	
			DR1(pAST2)/DR1	DR1(pAST2)-TC/DR1-TC
AOLE_09780	*fimA*	fimbrial family protein	−7.01	1.02
AOLE_06715	*fimC*	fimbrial protein	−5.69	−1.42
AOLE_09775	*fimD*	fimbrial protein	−5.66	1.51
AOLE_06720	*fimD*	fimbrial protein	−5.38	−1.80
AOLE_09770	*fimC*	chaperone protein mrkB	−4.94	−1.01
AOLE_03010	*fimT*	type IV fimbrial biogenesis protein FimT	−4.80	−4.19
AOLE_07050	*fimD*	fimbrial protein	−4.06	2.13
AOLE_16925	*pilF*	stability protein	−3.95	3.05
AOLE_11120	*fimC*	fimbrial chaperone	−3.29	−1.17
AOLE_17295	*fimV*	FimV domain-containing protein	−2.53	−1.15
AOLE_11125	*fimD*	fimbrial protein	−2.21	1.25

Membrane permeability was measured using 8-anilino-1-naphthylenesulfonic acid (ANS), a neutrally charged, hydrophobic probe that exhibits enhanced fluorescence in membrane-damaged cells [42], [43]. Membrane permeability is shown as red fluorescence intensity penetrating the cell membrane divided by µg protein (Figure 4C). Similar membrane permeability was observed in both wild-type and the *tetH* mutant strains. In contrast, the DR1 (pAST2) strain showed reduced permeability, presumably caused by cell membrane alteration by the TC resistant efflux pump. Under TC conditions, no significant differences were observed, although TetH deletion caused a slight increase in membrane permeability.

**Figure 5:**
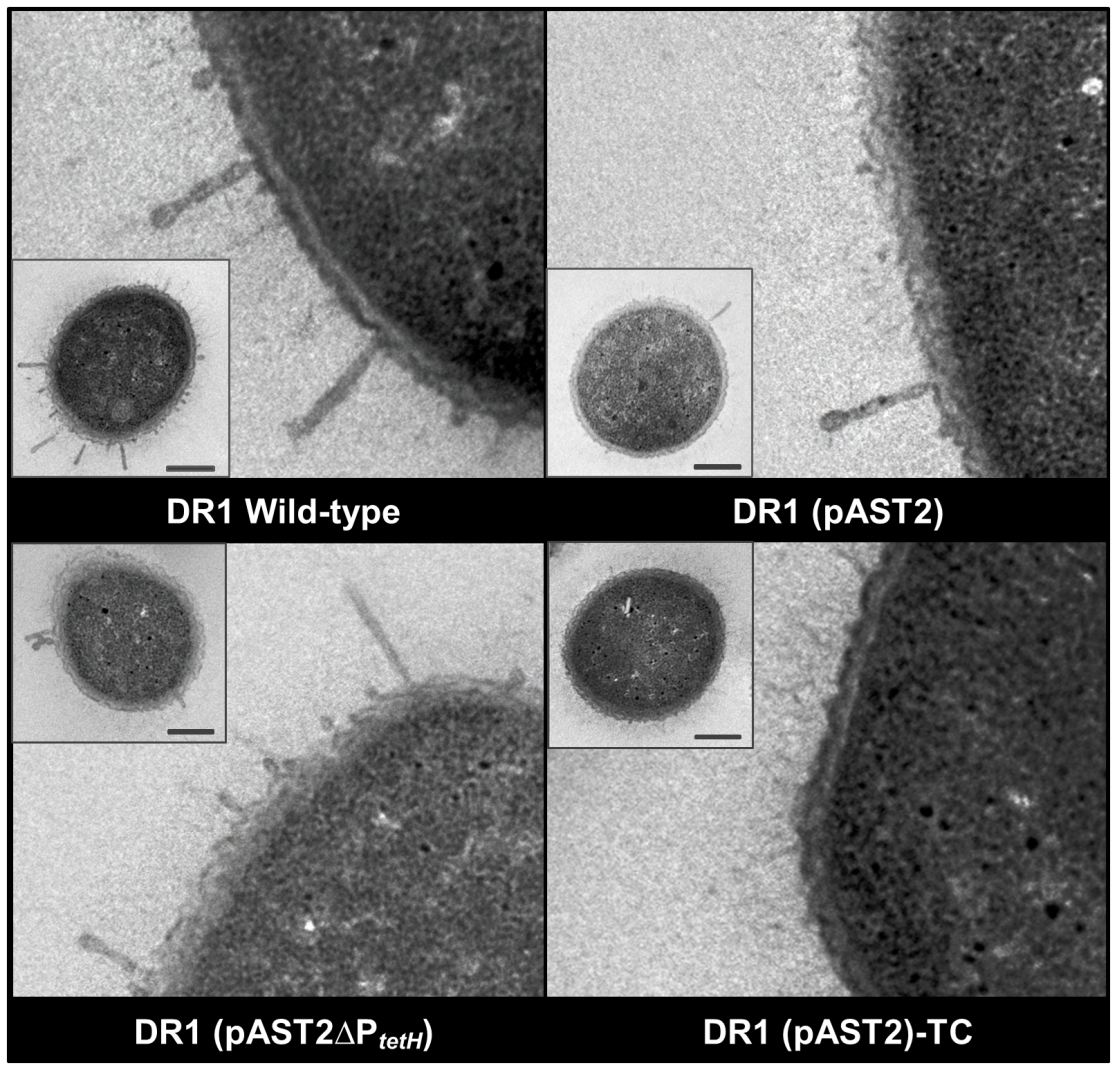
TEM analysis of DR1 strains. Microscopic images were obtained by transmission electron microscopy (TEM). Cells were cut into 70-nm-thick sections and double stained with uranyl acetate and lead citrate for contrast staining. The cross-section of each specimen is displayed in a small box alongside the longitudinal section. Bar indicates 250 nm.

Morphology of bacterial surface appendages was observed using transmission electron microscopy (TEM) (Figure 5). *A. oleivorans* DR1 cells have been reported to possess two major fimbrial appendages on the outer membrane [28], [41]. Thick fimbriae (28.2–29.3 nm in diameter) were sparsely distributed around the periphery of DR1 wild-type cells, and thin fimbriae (6.8–10 nm in diameter) were densely present on the membrane surface. Distribution of both types of fimbriae on the *tetH* mutant strain closely resembles that of wild-type cells; on DR1 cells harboring pAST2, thin fimbriae were absent but the thick fimbriae remained. Under TC conditions, the thick fimbriae were absent on DR1 (pAST2) cells, and only the thinner structures were observed. In addition, the presence of the pAST2 plasmid encoding the efflux pump increased membrane thickness from 50 nm to 60 nm in the absence of TC. However, a high level of membrane destruction was observed in both the pAST2-harboring strain and the *tetH* mutant strain.

### 6. Fitness cost of plasmid-mediated tetracycline resistance

**Figure 6:**
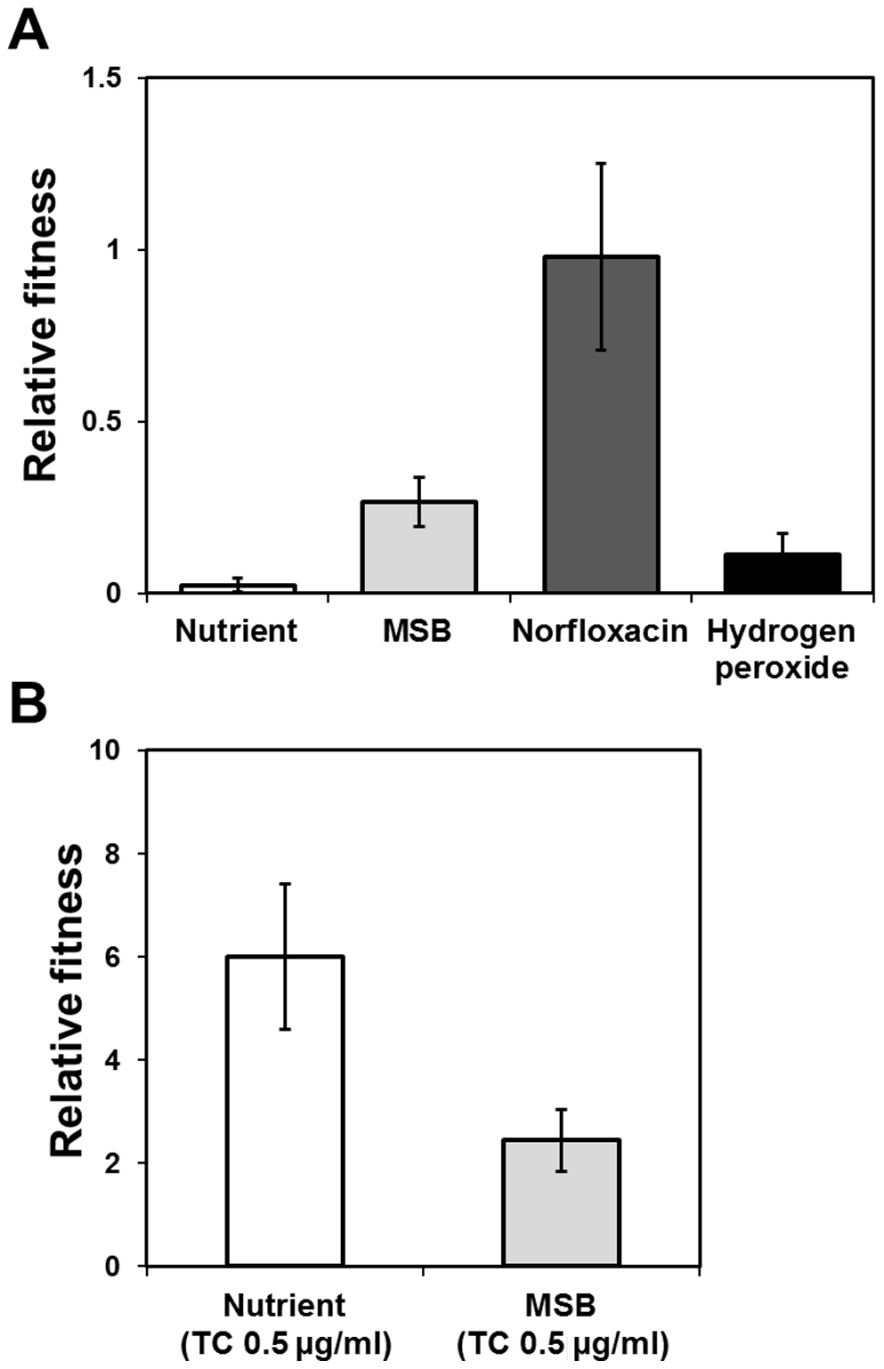
Fitness cost of plasmid-mediated tetracycline resistance. (A) Fitness by long-term survival of wild-type DR1 and DR1 (pAST2) in mixed cultures under test conditions. Norfloxacin and hydrogen peroxide were supplemented with sub-inhibitory concentrations in nutrient media. (B) Fitness under TC-treated conditions. The relative fitness is expressed as the ratio of DR1 (pAST2) to wild-type (fitness value; 1.0). Quantitative data were obtained from two independent mixed cultures.

A competition assay between the wild-type and plasmid-harboring strains was performed to measure fitness; growth was measured in nutrient media with or without antibiotics and oxidative compounds at sub-MIC concentrations (Figure 6). MSB medium was amended with 2% n-hexadecane to determine the competitive utilization of hexadecane. Relative fitness was expressed as the proportion with DR1 (pAST2) in co-culture cells. We found that the plasmid-harboring strain showed low fitness under non-antibiotic selective pressure (Figure 6A). This result was accordance with our observation that the growth rate of DR1 (pAST2) was much lower than that of the wild-type without TC. Interestingly, when norfloxacin was applied to the nutrient media, the plasmid-harboring strain possessed their proportion with equal to wild-type strain (0.98±0.27). Hydrogen peroxide (sub-MIC) applied in co-culture appeared to be uncompetitive (0.11±0.06). In contrast to this observation, peroxide stress at an above-MIC concentration provided resistance to the pAST2-carrying DR1 strain (Figure 3A). The plasmid-harboring strain showed 2.4 fold (MSB media) and 6 fold (nutrient broth) higher relative fitness in the presence of TC (Figure 6B). Our results suggest that plasmid-mediated TC resistance mitigated fitness costs in the presence of TC, whereas the plasmid-carrying population was considerably reduced under antibiotic-free conditions.

## Discussion

Bacteria gain antibiotic resistance by acquiring a plasmid encoding antibiotic resistance genes, but cells harboring the plasmid experience loss of fitness in the absence of antibiotic selective pressure [44]–[46]. Research on the influence of plasmid-mediated antibiotic resistance on bacterial physiology and fitness costs have primarily focused on growth defect [47]–[51]. Carriage of a plasmid yields clear benefits when the corresponding antibiotic is present [48], [52]. The fitness reduction observed in plasmid-carrying bacteria may contribute to the instability of plasmids in the environment because of competition with plasmid-free bacteria [53]. However, other studies have shown that expression of plasmid-encoded antibiotic resistance genes has an adverse effect on the reproductive fitness of plasmid-containing bacteria [54] and that harboring an antibiotic resistance plasmid triggered transcriptional deregulation [34]. Use of molecular machinery and energy for expressing plasmid genes in a host presumably alters expression of host genes. Thus, many other phenotypes may be modulated by possession of a plasmid. Elimination of resistance genes from a plasmid can lower the fitness burden on host bacteria [55], [56]. In addition, repression of a resistance gene can be effective in avoiding the cost of resistance in an antibiotic-free environment [57].

*Acinetobacter* species carrying a resistance plasmid showed decreased fitness in the absence of antibiotics [58]. We have also shown a relationship between bacterial fitness costs and antibiotic resistance in *Acinetobacter oleivorans* DR1 [27], [40]. Acquisition of the extracellular plasmid pAST2 altered phenotypes, and the phenotypic changes are thought to be linked to changes in host gene expression [29]. In order to gain further insight into the phenotypic changes caused by uptake of extracellular genetic material, we performed an RNA-Seq analysis of the entire transcriptome. To validate our RNA-Seq result, quantitative real-time PCR (qRT-PCR) confirmed the gene expression of 10 genes selected based on category and expression value (Figure S4). The results showed that the expression values of those genes were closely matched to RNA-Seq data (Figure S4). Our findings showed that all plasmid-encoded genes were highly expressed, which altered not only host gene expression, but caused phenotypic and physiological changes. Interestingly, our transcriptomic data showed that many membrane-related genes and most fimbrial proteins encoded by *fim* genes were considerably downregulated (Table 4, Table S2). In contrast, membrane appendage pilin-related genes were highly upregulated [e.g. *pilA* (AOLE_01545, 218.7-fold increase), data not shown] by possession of the plasmid. However, we did not observe pili in our TEM analysis (Figure 5). We are unable to explain this discrepancy, but hypothesize that the pilus may be lost during preparation of TEM images. It is interesting to note that the natural competence-associated type IV pilus assembly protein encoded by AOLE_15230 (3.5-fold increase) and AOLE_17785 (3.69-fold increase) were also upregulated, and plasmid-mediated horizontal gene transfer by expression of pilus genes merits investigation in this strain. The low levels of fimbriae expression observed may be linked to loss of adhesion to hexadecane droplets, reduced membrane permeability, or diminished motility [40].

Membrane integrity and permeability may play important roles in many bacterial stress responses [59], [60]. Our data demonstrated for the first time that, in addition to the plasmid replication burden, the plasmid-encoded membrane bound efflux pump encoded by *tetH* gene is important for altering bacterial physiology and phenotypes such as peroxide resistance, membrane permeability, and fimbria expression. Physical incorporation of the efflux pump into the membrane appears to be critical for altering membrane integrity. The efflux pump protein encoded by TetH in pAST2 contains 400 amino acids with an unusual 11 transmembrane domains; most tetracycline efflux pumps, such as TetA, contain 12 transmembrane helices (data not shown). The TetH protein belongs to the major facilitator superfamily (MFS) of transporters, which has little specificity [61]. In many bacterial genomes, membrane-transport proteins comprise only 0.1–1.0% of the total proteins, and the expression of many membrane proteins is very low (less than 0.1% of total cell proteins) under typical conditions [62]. Our data demonstrated that high expression of the plasmid-encoded TetH altered the physiological status of cells, including hydrogen peroxide resistance, reduction of motility, and altered fimbria expression. Modulation of biological fitness by the plasmid-mediated tetracycline resistance TetH efflux pump may be attributed to the interference of the plasmid-encoded membrane-bound tetracycline efflux pump. In conclusion, the significant alteration of bacterial membrane integrity caused by the plasmid-mediated efflux pump affects bacterial phenotype and biological fitness in the environment.

## Materials and Methods

### 1. Bacterial strains and growth conditions

The bacterial strains, plasmids, and primers (from 5′ to 3′) utilized in this study are listed in Table S3. The hexadecane-degrading bacterium, *Acinetobacter oleivorans* DR1, was grown at 30°C in nutrient broth (Marine BioProducts, Canada) and shaken at 220 rpm for aeration. DR1 strains harboring plasmids were grown under the same conditions as the wild-type DR1 strain. The *E. coli* strains were grown at 37°C in LB broth and shaken at 220 rpm for aeration. The tetracycline-resistance plasmid pAST2 that was transformed into strain DR1 used in this study was environmentally isolated, as described previously [29]. The complete sequence of the plasmid was deposited in the National Center for Biotechnology Information (NCBI) database under accession number KC734561. The genome sequence of *A. oleivorans* DR1 was retrieved from the NCBI database (accession number NC_014259.1).

### 2. RNA-Seq analysis

Strain DR1 carrying pAST2 and wild-type strain DR1 were grown overnight at 30°C with shaking at 220 rpm. A 50 µl stationary-phase culture was used to inoculate 5 ml fresh nutrient medium and then incubated under the same conditions. Each strain was grown to the exponential phase (OD_600_ of ∼0.4) at 30°C in nutrient medium supplemented with or without 1 µg/ml tetracycline over a period of 15 min. Total RNA was extracted using an RNeasy Mini Kit (Qiagen, USA), following the manufacturer’s instructions. All procedures for RNA sequencing and alignment were carried out by Chunlab (Seoul, South Korea). RNA was subjected to a subtractive Hyb-based rRNA removal process using the MICROBExpress Bacterial mRNA Enrichment Kit (Ambion, USA). Subsequent processing, including library construction, was conducted as described previously [63]. RNA sequencing was performed using two runs of the Illumina Genome Analyzer IIx to generate single-ended 36-bp reads. Quality-filtered reads were aligned to the *A. oleivorans* DR1 genome as a reference sequence by using the CLC Genomics Workbench 4.0 (CLC Bio, Denmark). Mapping was based on a minimal length of 32 bp with an allowance of up to 2 mismatches. The relative transcript abundance was measured in reads per kilobase of exon sequence per million mapped sequence reads (RPKM) [64]. The mapping results were visualized using the CLRNA-Seq programs (Chunlab, South Korea). The fold-change [RPKM of DR1 (pAST2)/RPKM of DR1, RPKM of DR1-TC/RPKM of DR1, RPKM of DR1 (pAST2)-TC/RPKM of DR1 (pAST2), and RPKM of DR1 (pAST2)-TC/RPKM of DR1-TC] represented the level of gene up- or downregulation. Genes showing fold-changes larger than 2.0 were designated as upregulated, and those showing fold-changes greater than −2.0 were designated as downregulated. The RNA-Seq data were deposited in the National Center for Biotechnology Information (NCBI) GEO site under accession numbers GSE38340, GSE44428, and GSE55239.

### 3. Gene expression analysis using qRT-PCR

Wild-type DR1 and DR1(pAST2) were grown to the exponential growth phase (OD_600_ = ∼0.4) at 30°C in nutrient medium. The exponentially growing cells (OD_600_ = ∼0.4) were treated with 1 µg/ml TC and then incubated for 15 min. Total RNA was extracted from 5 ml of cultures using an RNeasy Mini kit (Qiagen, Valencia, CA, USA) according to the manufacturer’s instructions. cDNA was synthesized from 1 µg RNA by using gene specific primers based on sequence of *A. oleivorans* genome (Table S3) and the primers for genes were used as templates for quantitative real-time PCR (qRT-PCR). The 25 µl PCR mixture included 12.5 µl iQ SYBR Green Supermix (Bio-Rad), 2 µl of each primer (0.5 µM), 3 µl cDNA, and 5.5 µl distilled water. The PCR conditions were 95°C for 3 min, followed by 35 cycles consisting of 23 s at 95°C, 23 s at 60°C, and 23 s at 72°C. The expression level of each gene was normalized to the 16S rRNA expression level that was quantified with 16s rRNA-341F/16s rRNA-534R primers. Relative quantifications were performed in triplicate.

### 4. Construction of the *tetH-tetR* mutant, DR1 (pAST2ΔP_tetH_)

The suicide vector pCVD442 [65] was used to construct a *tetH-tetR* mutant. Polymerase chain reaction (PCR) and gel electrophoresis were carried out as described previously [66]. A 463-bp fragment of the internal region of the *tetH* gene that had been amplified using *tetH*-F/*tetH*-R primers was cloned into the *Kpn*I/*Bam*HI cloning sites of the pBBR1MCS4 vector, generating pBBR1MCS4-*tetH* (Figure S5). The plasmid was extracted using a Dyne Plasmid Miniprep Kit (DYNEBIO, Korea). The amplicon (440 bp) of the partial region of the *tetR* gene obtained using the *tetR*-F/*tetR*-R primer pair was cloned into the *Bam*HI/*Sac*I cloning sites of the pBBR1MCS4-*tetH* vector to yield pBBR1MCS4-*tetHtetR*. A full-length kanamycin cassette (1,264 bp) from the plasmid pUC4K was digested with *Bam*HI and cloned into the pBBR1MCS4-*tetHtetR* vector to obtain pBBR1MCS4-*tetHtetR*::*km*. The constructed plasmid was digested using *Kpn*I/*Sac*I (Thermo Fisher Scientific, USA) to obtain the *tetHtetR*::*km* fragment and finally cloned into the pCVD442 vector to yield pCVD442-*tetHtetR*::*km*. The constructed plasmid was then introduced into *A. oleivorans* DR1 (pAST2) by electroporation. Transformation was performed with 2.5 µl of plasmid DNA and 50 µl *A. oleivorans* DR1 (pAST2) competent cells using a micropulser (Bio-Rad, USA) with a time constant range of 4.0–5.0 ms and a constant voltage of 5.5–6 kV. PCR was conducted to confirm the inserted fragments using the *tetH*-F/*tetR*-R primer set (Table S3).

### 5. Bioluminescent ATP assay

Intracellular ATP concentrations were measured using the ENLITEN ATP Assay System Bioluminescence Detection Kit (Promega, USA). Exponentially grown cells (OD_600_ of ∼0.4) were treated with 0.5 µg/ml TC or without TC for 15 min and cells were harvested by centrifugation (13,000×*g*, 1 min). The pellet was suspended with 1% trichloroacetic acid (TCA) buffer for ATP extraction. Each sample in TCA buffer was diluted 5 fold with Tris-acetate buffer to neutralize the extracts. The sample (20 µl) was then mixed with 100 µl of luciferin–luciferase reagent, and the bioluminescence intensity, in relative light units (RLU), was recorded immediately using a microtiter plate reader (VICTOR^3^, BioRad, USA) with 10 s integration time. An ATP standard curve was constructed in accordance with the manufacturer’s instructions (Figure S6). The ATP concentration was calculated as molar concentration per mg of protein. ATP assays were performed from triplicate experiments. Statistical significance of differences was calculated using Student’s *t*-test.

### 6. Hexadecane degradation and microbial adherence to *n*-hexadecane (MATH)

*A. oleivorans* DR1 strains were inoculated at ∼10^6^ CFU/ml in 20 ml of MSB media supplemented with 2% (v/v) *n*-hexadecane (Sigma, USA). Degradation of hexadecane was monitored by measuring the OD_600_ of each strain. After TC (0.5 µg/ml) was added to the exponentially grown cells (OD_600_ of ∼0.4) and they were incubated for 30 min, 2 ml of bacterial cells were harvested by centrifugation (13,000×*g*, 1 min) and washed twice with phosphate-buffered saline (PBS). The OD_600_ of the resuspended cells was measured in the same way as the initial OD. The cells were agitated with 200 µl of *n*-hexadecane using a vortexer for 10 min. After 15 min of immobilization, the mixed solution was divided into two separate phases. We measured the absorbance of the aqueous layer at 600 nm as the final OD. The hydrophobicity of each strain was calculated as the final OD divided by the initial OD, expressed as a percentage (%). The quantitative data were obtained from triplicate experiments. Statistical significance of differences was calculated using Student’s *t*-test.

### 7. Sensitivity of *Acinetobacter* strains to antibiotics

*A. oleivorans* DR1 strains were grown in nutrient broth with shaking at 30°C. The stationary-grown cells were diluted 100 fold in 5 ml of fresh media and incubated until the OD_600_ of the cells reached the exponential phase (OD_600_ of ∼0.4). The exponentially growing cells were harvested by centrifugation (13,000×*g*, 1 min) and washed twice with PBS. One hundred-microliter samples containing 10^5^ cells were serially diluted to 10^4^, 10^3^,10^2^, 10^1^, and 10^0^ with 900 µl PBS. Each 5 µl of diluted solution was spotted on a plate and incubated at 30°C for 12 h. Antibiotics were amended at sub-MIC levels on nutrient agar supplemented with norfloxacin (Nor, 2 µg/ml), ampicillin (Amp, 25 µg/ml), gentamicin (Gm, 0.2 µg/ml), kanamycin (Km, 0.5 µg/ml), rifampicin (Rif, 4 µg/ml), or chloramphenicol (32 µg/ml).

### 8. Bacterial inhibition assay to determine sensitivity to oxidative stress

A bacterial inhibition assay was conducted to determine the sensitivity of *Acinetobacter* strains to oxidative stress. Bacterial cell growth was monitored by measuring the OD_600_ of the cultures using a Biophotometer (Eppendorf, Germany). When the bacterial cell cultures reached the exponential phase (OD_600_ of ∼0.4), 5 ml of washed cell culture (with PBS) was mixed with 25 ml of autoclaved nutrient agar. Autoclaved paper disks (8×0.7 mm, ADVANTEC, Japan) were placed on the plate and 25 µl of each oxidative compound was added to a final concentration of 1 M hydroperoxide (H_2_O_2_), 1 M cumene hydroperoxide (CHP), 100 mM menadione (MD), and 100 mM paraquat (PQ). After incubation at 30°C for 24 h, the clear zones were evaluated by measuring their diameter (mm). All quantitative data were obtained from two independent cultures.

### 9. Swimming and swarming motility

Swimming and swarming motility were tested on 0.2% and 0.5% agar-containing medium, respectively. *A. oleivorans* DR1 strains were grown to the exponential growth phase (OD_600_ of ∼0.4) and 1 µl of ∼10^9^ cell culture was spotted onto nutrient media. After 24 h and 72 h incubation at 30°C, bacterial motilities were evaluated by measuring their diameter (mm). All quantitative data were obtained in triplicate.

### 10. Cell membrane permeability assays

A fluorescent probe, 8-anilino-1-naphthylenesulfonic acid (ANS; Sigma-Aldrich, USA), was used to assess the integrity of bacterial cell membranes. Overnight cultures were diluted 100 fold in 5 ml fresh medium and grown to the logarithmic growth phase at 30°C with shaking at 220 rpm. After the cells were treated with or without 0.5 µg/ml TC at the exponential phase (OD_600_ = ∼0.4) for 30 min, a 1-ml cell culture was harvested by centrifugation (13,000×*g*, 1 min) and washed twice with PBS. The resuspended solutions were supplemented with ANS (1 µl, 3 mM) at room temperature for 10 min in the dark. The fluorescence intensity of cells was quantified using a microplate reader. The filter set for fluorescence included a 555 nm excitation filter and 590 nm emission filter. The possibility of different growth rates under the experimental conditions was excluded by normalizing µg protein. Cell membrane permeability assays were performed three times independently. Statistical significance of differences was calculated using Student’s *t*-test.

### 11. Transmission electron microscopy

Overnight cultures were diluted 100 fold in 5 ml fresh medium and grown to the logarithmic growth phase at 30°C with shaking at 220 rpm. A 1-ml cell culture was harvested by centrifugation (10,000×*g*, 1 min) and was washed twice with PBS. Each sample was fixed with Karnovsky’s fixative containing 0.5% calcium chloride (Sigma-Aldrich, USA), 2% glutaraldehyde (MERCK, Germany), and 2% paraformaldehyde (MERCK, Germany) in 0.1 M phosphate buffer (pH 7.4) for 2 h and washed three times. Samples were post-fixed with 1% osmium tetroxide (OsO_4_; Polysciences, USA) dissolved in 0.1 M PB for 2 h and dehydrated in a gradually ascending ethanol series (50–100%) and infiltrated with propylene oxide. Specimens were embedded using a Poly/Bed 812 kit (Polysciences, USA). After embedding in pure fresh resin and polymerization at 60°C in an electron microscope oven (TD-700, DOSAKA, Japan) for 24 h, 350-nm-thick sections were cut and stained with toluidine blue for light microscopy. Seventy-nanometer-thick sections were double stained with 7% (20 min) uranyl acetate and lead citrate for contrast staining. Sections were cut using a LEICA EM UC-7 Ultra-microtome (Leica Microsystems, Austria). All the thin sections were observed using a transmission electron microscope (JEM-1011, 80Kv JEOL, Japan) at an acceleration voltage of 80 kV.

### 12. Growth competition assay

A competition assay between the wild-type and plasmid-harboring strains was performed to determine growth fitness; cells were grown in nutrient media with or without tetracycline (0.5 µg/ml), hydrogen peroxide (0.15 mM), and norfloxacin (2 µg/ml) or MSB media supplemented with 2% n-hexadecane. Two exponentially grown cell cultures (OD_600_ of ∼0.4) were washed twice with PBS and ∼10^6^ CFU/ml of each cell type was mixed in the appropriate media. After 3 days of cultivation at 30°C with shaking at 220 rpm, relative fitness was determined by measuring the total number of viable cells on nutrient agar or selective plates containing TC for wild-type DR1 or DR1 (pAST2), respectively. The relative fitness was calculated using the following equation [Colony number of antibiotics variants/(Total viable cells - colony number of antibiotics variant)]. Quantitative data were obtained from two independent mixed cultures.

## Supporting Information

Figure S1 **An xy plot of RPKM values from DR1 strains grown on nutrient with or without TC (MIC, 1 µg/ml).** A dot indicates a gene, and its x and y coordinates indicate the RPKM from the following data sets. (A) DR1 (pAST2)/DR1; (B) DR1 (pAST2)-TC/DR1-TC; (C) DR1 (pAST2)-TC/DR1 (pAST2); (D) DR1-TC/DR1. Fold changes (RPKM ratio) are represented with a color gradient. Red dots indicate genes upregulated more than 2 fold. Levels of gene expression (−2<fold change values<2) are shown in three different colors. Orange dots indicate 1.5≤fold change values of<2, gray dots show −1.5<fold change values of<1.5, and blue dots indicate −2<fold change values of≤−1.5. Dark blue dots indicate genes downregulated less than 2 fold.
(TIF)

Figure S2 **Clusters of orthologous groups (COGs) assignments of differently expressed genes.** The number of upregulated and downregulated genes was sorted according to COGs. Colors of the bars indicate the fold change in gene expression. Red, gene expression with more than a 2-fold change in value; Gray, gene expression with between a −2 and 2-fold change in value; Blue, gene expression with less than a −2-fold change in value. One-letter abbreviations for functional categories: A, RNA processing and modification; C, energy production and conversion; D, cell cycle control and mitosis; E, amino acid metabolism and transport; F, nucleotide metabolism and transport; G, carbohydrate metabolism and transport; H, coenzyme metabolism; I, lipid metabolism; J, translation, including ribosome structure and biogenesis; K, transcription; L, replication, recombination, and repair; M, cell wall structure, biogenesis, and outer membrane; N, secretion, motility, and chemotaxis; O, molecular chaperones and related functions; P, inorganic ion transport and metabolism; Q, secondary metabolite biosynthesis, transport, and catabolism; T, signal transduction; U, intracellular trafficking, secretion, and vesicular transport; and V, defense mechanisms. (A) DR1 (pAST2)/DR1; (B) DR1 (pAST2)-TC/DR1-TC; (C) DR1 (pAST2)-TC/DR1 (pAST2); (D) DR1-TC/DR1.
(TIF)

Figure S3 **Sensitivity of DR1 strains to six antibiotics with or without TC supplementation.** (A) Sensitivity of DR1 strains to six antibiotics in control nutrient media. (B) Sensitivity of DR1 strains to six antibiotics with sub-MIC levels of TC (0.5 µg/ml). Antibiotics were amended at sub-MIC levels. Nutrient media was supplemented with norfloxacin (Nor, 2 µg/ml), ampicillin (Amp, 25 µg/ml), gentamicin (Gm, 0.2 µg/ml), kanamycin (Km, 0.5 µg/ml), rifampicin (Rif, 4 µg/ml), or chloramphenicol (32 µg/ml).
(TIF)

Figure S4 **Confirmation of gene expression data from RNA-Seq using qRT-PCR.** Ten genes were selected based on category and expression value. Error bar of qRT-PCR data was obtained from triplicate experiments (A) Fold change of DR1(pAST2)/DR1 (B) Fold change of DR1(pAST2)-TC/DR1-TC.
(TIF)

Figure S5 **Construction of** ***tetH*** **efflux pump mutant strain, DR1 (pAST2ΔP** ***_tetH_*** **).** (A) *tetH*-*tetR* gene disruption strategy. Deletion of the intergenic region between *tetH* and *tetR* was performed according to the following method. First, a partial *tetH* gene (463 bp) was cloned into the ampicillin-marked shuttle vector. Second, internal *tetR* (440 bp) was inserted into the constructed vector near the *tetH* gene. Third, a full-length kanamycin cassette (1264 bp) from the plasmid pUC4K was inserted between *tetH* and *tetR*. The cloned fragment, *tetHtetR*::*km*, was inserted into the suicide vector and finally transformed into DR1 (pAST2). (B) PCR verification of recombinant strains using the *tetH*-F/*tetR*-F primer pair. M, 1-kb DNA ladder (Fermentas); 1, *E. coli* Top10 control; 2, DR1 wild-type; 3, *E. coli* Top10 (pBBR1MCS4-*tetHtetR*), 903 bp; 4, DR1 (pAST2), 1326 bp; 5, *E. coli* Top10 (pBBR1MCS4-*tetHtetR*::*km*), 2167 bp; 6, DR1 (pAST2ΔP*_tetH_*), 2167 bp. (C) PCR verification of mutant plasmid (pAST2ΔP*_tetH_*) construction using the *tetH*-F/*tetR*-F primer pair. M, 1-kb DNA ladder (Fermentas); 1, plasmid DNA of pAST2 (1326 bp); 2, plasmid DNA of pCVD442-*tetHtetR*::*km* (2167 bp); 3, plasmid DNA of pAST2ΔP*_tetH_* (2167 bp).
(TIF)

Figure S6 **Standard curves were constructed using relative light units (RLU) and known ATP concentrations.**
(TIF)

Table S1 **Antibiotic resistance and oxidative stress-related gene expression profiles.**
(DOC)

Table S2 **Outer membrane-related gene expression profiles.**
(DOC)

Table S3 **Bacterial strains, plasmids, and primers used in this study.**
(DOC)
