# Supplementary figures and images for: Plasmid-Encoded Tetracycline Efflux Pump Protein Alters Bacterial Stress Responses and Ecological Fitness of Acinetobacter oleivorans
Source: PLoS One. 2014 Sep 17;9(9):e107716. doi: 10.1371/journal.pone.0107716 (PMC4167995; doi:10.1371/journal.pone.0107716)

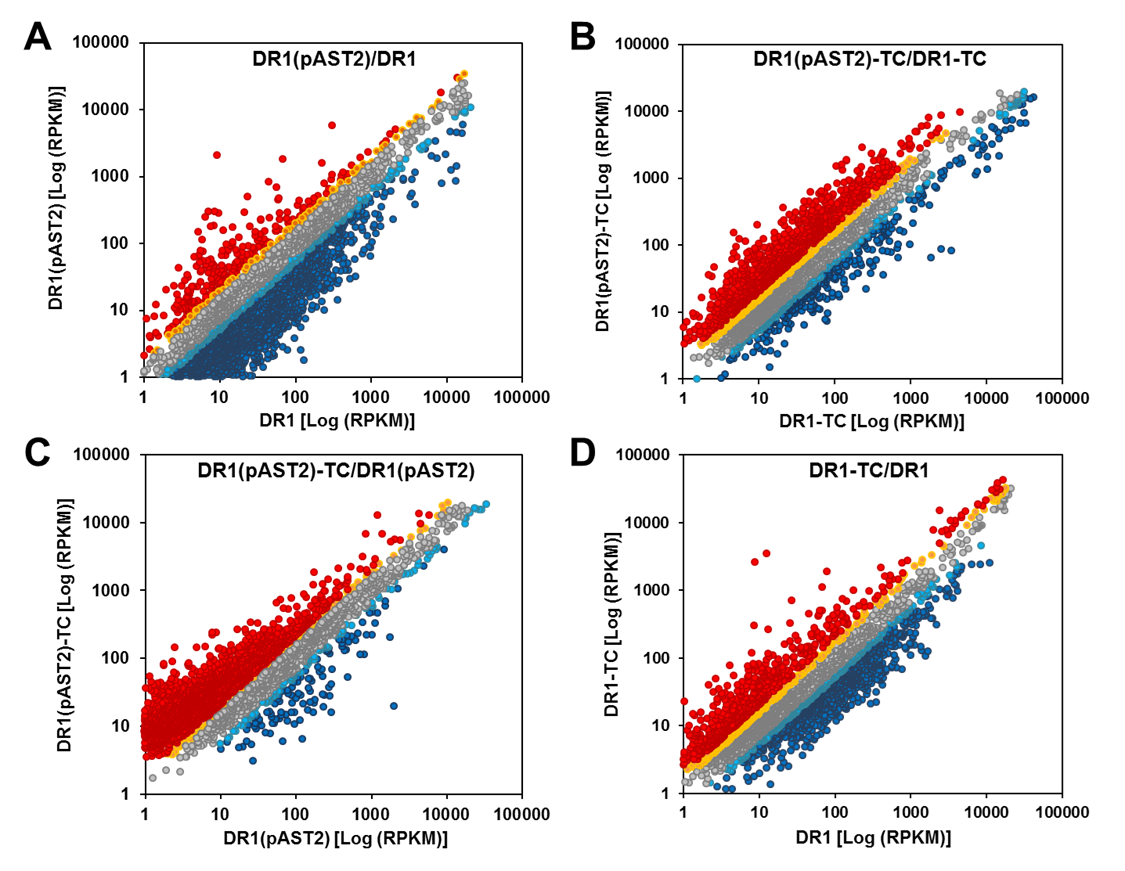

Supplement: Figure S1 — An xy plot of RPKM values from DR1 strains grown on nutrient with or without TC (MIC, 1 µg/ml). A dot indicates a gene, and its x and y coordinates indicate the RPKM from the following data sets. (A) DR1 (pAST2)/DR1; (B) DR1 (pAST2)-TC/DR1-TC; (C) DR1 (pAST2)-TC/DR1 (pAST2); (D) DR1-TC/DR1. Fold changes (RPKM ratio) are represented with a color gradient. Red dots indicate genes upregulated more than 2 fold. Levels of gene expression (−2<fold change values<2) are shown in three different colors. Orange dots indicate 1.5≤fold change values of<2, gray dots show −1.5<fold change values of<1.5, and blue dots indicate −2<fold change values of≤−1.5. Dark blue dots indicate genes downregulated less than 2 fold. (TIF) [file pone.0107716.s001.tif]

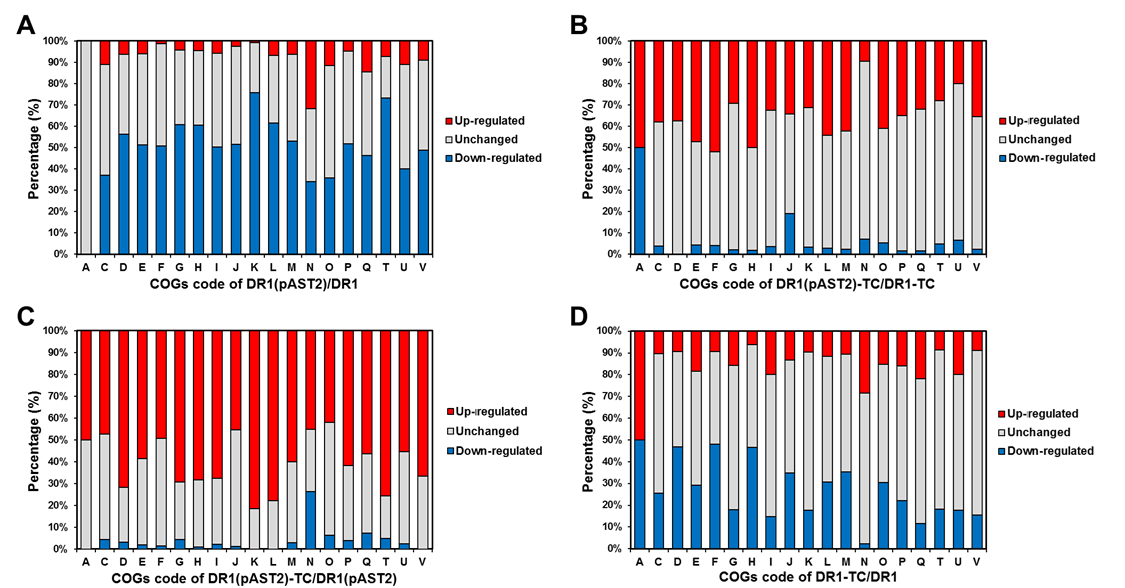

Supplement: Figure S2 — Clusters of orthologous groups (COGs) assignments of differently expressed genes. The number of upregulated and downregulated genes was sorted according to COGs. Colors of the bars indicate the fold change in gene expression. Red, gene expression with more than a 2-fold change in value; Gray, gene expression with between a −2 and 2-fold change in value; Blue, gene expression with less than a −2-fold change in value. One-letter abbreviations for functional categories: A, RNA processing and modification; C, energy production and conversion; D, cell cycle control and mitosis; E, amino acid metabolism and transport; F, nucleotide metabolism and transport; G, carbohydrate metabolism and transport; H, coenzyme metabolism; I, lipid metabolism; J, translation, including ribosome structure and biogenesis; K, transcription; L, replication, recombination, and repair; M, cell wall structure, biogenesis, and outer membrane; N, secretion, motility, and chemotaxis; O, molecular chaperones and related functions; P, inorganic ion transport and metabolism; Q, secondary metabolite biosynthesis, transport, and catabolism; T, signal transduction; U, intracellular trafficking, secretion, and vesicular transport; and V, defense mechanisms. (A) DR1 (pAST2)/DR1; (B) DR1 (pAST2)-TC/DR1-TC; (C) DR1 (pAST2)-TC/DR1 (pAST2); (D) DR1-TC/DR1. (TIF) [file pone.0107716.s002.tif]

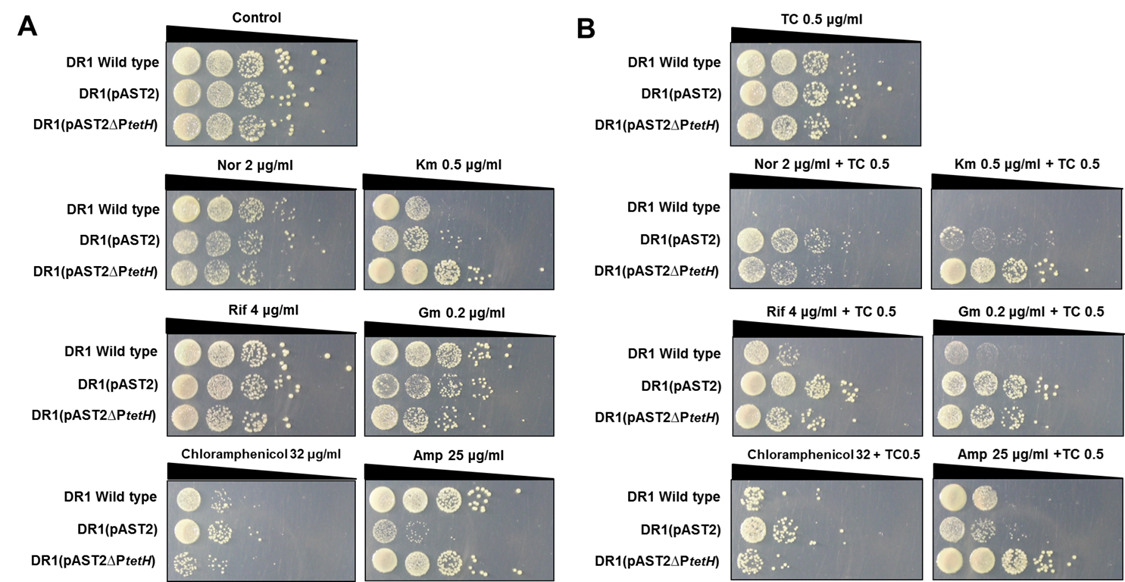

Supplement: Figure S3 — Sensitivity of DR1 strains to six antibiotics with or without TC supplementation. (A) Sensitivity of DR1 strains to six antibiotics in control nutrient media. (B) Sensitivity of DR1 strains to six antibiotics with sub-MIC levels of TC (0.5 µg/ml). Antibiotics were amended at sub-MIC levels. Nutrient media was supplemented with norfloxacin (Nor, 2 µg/ml), ampicillin (Amp, 25 µg/ml), gentamicin (Gm, 0.2 µg/ml), kanamycin (Km, 0.5 µg/ml), rifampicin (Rif, 4 µg/ml), or chloramphenicol (32 µg/ml). (TIF) [file pone.0107716.s003.tif]

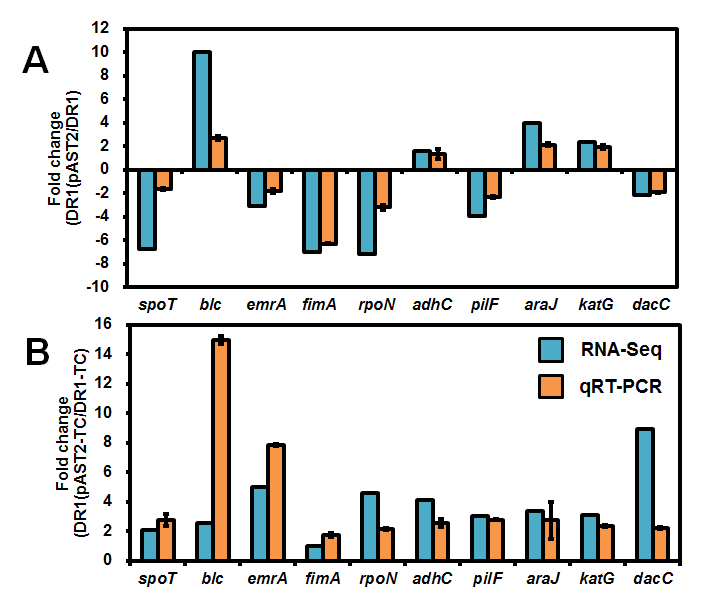

Supplement: Figure S4 — Confirmation of gene expression data from RNA-Seq using qRT-PCR. Ten genes were selected based on category and expression value. Error bar of qRT-PCR data was obtained from triplicate experiments (A) Fold change of DR1(pAST2)/DR1 (B) Fold change of DR1(pAST2)-TC/DR1-TC. (TIF) [file pone.0107716.s004.tif]

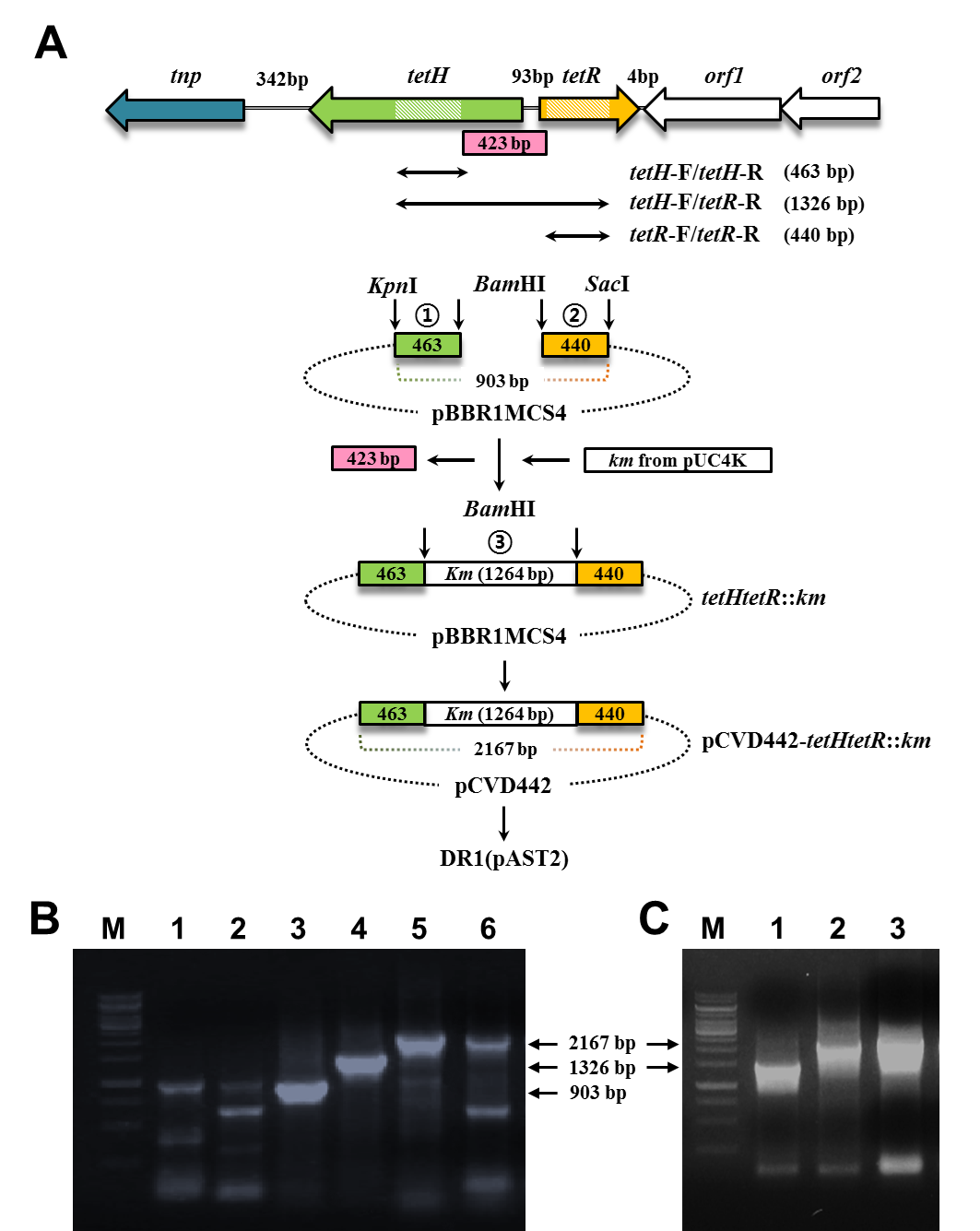

Supplement: Figure S5 — Construction of tetH efflux pump mutant strain, DR1 (pAST2ΔP tetH ). (A) tetH-tetR gene disruption strategy. Deletion of the intergenic region between tetH and tetR was performed according to the following method. First, a partial tetH gene (463 bp) was cloned into the ampicillin-marked shuttle vector. Second, internal tetR (440 bp) was inserted into the constructed vector near the tetH gene. Third, a full-length kanamycin cassette (1264 bp) from the plasmid pUC4K was inserted between tetH and tetR. The cloned fragment, tetHtetR::km, was inserted into the suicide vector and finally transformed into DR1 (pAST2). (B) PCR verification of recombinant strains using the tetH-F/tetR-F primer pair. M, 1-kb DNA ladder (Fermentas); 1, E. coli Top10 control; 2, DR1 wild-type; 3, E. coli Top10 (pBBR1MCS4-tetHtetR), 903 bp; 4, DR1 (pAST2), 1326 bp; 5, E. coli Top10 (pBBR1MCS4-tetHtetR::km), 2167 bp; 6, DR1 (pAST2ΔPtetH), 2167 bp. (C) PCR verification of mutant plasmid (pAST2ΔPtetH) construction using the tetH-F/tetR-F primer pair. M, 1-kb DNA ladder (Fermentas); 1, plasmid DNA of pAST2 (1326 bp); 2, plasmid DNA of pCVD442-tetHtetR::km (2167 bp); 3, plasmid DNA of pAST2ΔPtetH (2167 bp). (TIF) [file pone.0107716.s005.tif]

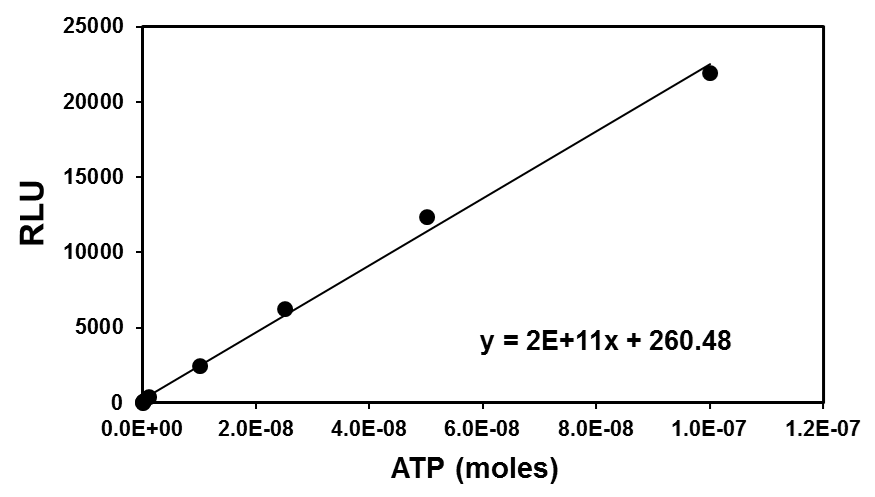

Supplement: Figure S6 — Standard curves were constructed using relative light units (RLU) and known ATP concentrations. (TIF) [file pone.0107716.s006.tif]
