# Supplementary material for: Plasmid-Encoded Tetracycline Efflux Pump Protein Alters Bacterial Stress Responses and Ecological Fitness of Acinetobacter oleivorans
Source: PLoS One. 2014 Sep 17;9(9):e107716. doi: 10.1371/journal.pone.0107716 (PMC4167995; doi:10.1371/journal.pone.0107716)
Supplement: Table S1 — Antibiotic resistance and oxidative stress-related gene expression profiles. (DOC) [file pone.0107716.s007.doc]

**Table S1. Antibiotic resistance and oxidative stress-related gene expression profiles.**

| **Locus_tag** | **Gene** | **Product** | **Fold change** | |
| --- | --- | --- | --- | --- |
|  |  |  | **DR1(pAST2)/DR1** | **DR1(pAST2)-TC/DR1-TC** |
| **Antibiotic resistance-related genes** | | | | |
| AOLE_02025 | *adeT* | putative aminoglycoside resistance efflux pump | −11.86 | 1.22 |
| AOLE_11070 | *ampC* | beta-lactamase class D | −11.68 | 2.37 |
| AOLE_09290 | *araJ* | drug resistance MFS transporter | −10.15 | 1.73 |
| AOLE_12985 | *wbbJ* | chloramphenicol acetyltransferase | −8.77 | 1.00 |
| AOLE_05220 | *ampC* | beta-lactamase | −7.14 | 2.50 |
| AOLE_18125 | *tolC* | channel-tunnel spanning the outer membrane | −6.84 | 2.14 |
| AOLE_17460 | *norM* | multidrug ABC transporter | −5.73 | −1.20 |
| AOLE_00050 | *dctP* | RND type efflux pump | −5.16 | −1.67 |
| AOLE_09410 | - | RND type efflux pump | −3.70 | 1.23 |
| AOLE_09285 | *emrA* | multidrug resistance efflux pump | −3.08 | 5.03 |
| AOLE_09305 | *tolC* | outer membrane protein tolC | −3.07 | 2.06 |
| AOLE_03130 | *acrB* | nodulation protein | −2.54 | 1.21 |
| AOLE_03795 | *wbbJ* | chloramphenicol acetyltransferase | −2.45 | 1.30 |
| AOLE_05885 | *tolC* | RND efflux system, outer membrane lipoprotein,NodT | −2.33 | 1.52 |
| AOLE_00140 | *acrA* | putative RND efflux membrane fusion protein | 2.54 | 1.61 |
| AOLE_15165 | *emrA* | multidrug resistance protein A | 2.67 | 3.71 |
| AOLE_00145 | *acrB* | AcrB/AcrD/AcrF family protein | 3.12 | 1.74 |
| AOLE_15160 | *araJ* | multidrug resistance protein B | 3.95 | 3.39 |
| **Oxidative stress-related genes** | | | | |
| AOLE_01575 | *spoT* | GTP pyrophosphokinase (ppGpp synthetase I) | −6.76 | 2.12 |
| AOLE_12135 | *soxR* | redox-sensitive transcriptional activator SoxR | −6.08 | −1.20 |
| AOLE_18445 | - | SoxR family transcriptional regulator | −3.94 | 1.43 |
| AOLE_01750 | *sodC* | Cu/Zn superoxide dismutase | −1.94 | 1.10 |
| AOLE_16785 | *grxC* | glutaredoxin 3 | −1.84 | 1.49 |
| AOLE_16430 | - | thioredoxin | −1.73 | 1.37 |
| AOLE_13410 | *ahpF* | alkyl hydroperoxide reductase subunit F | −1.56 | −1.74 |
| AOLE_16635 | *tdcF* | hypothetical protein | −1.26 | −1.22 |
| AOLE_15340 | *trxB* | thioredoxin-disulfide reductase | −1.18 | 3.74 |
| AOLE_14380 | *OxyR* | Hydrogen peroxide-inducible genes activator | −1.09 | 3.02 |
| AOLE_19220 | *frnE* | Thiol-disulfide isomerase and thioredoxin | 1.14 | 1.10 |
| AOLE_09800 | *katE* | hypothetical protein | 1.16 | 1.51 |
| AOLE_14365 | - | rubredoxin | 1.19 | −1.55 |
| AOLE_02585 | - | thioredoxin 2 | 1.46 | 1.35 |
| AOLE_05305 | *sodA* | superoxide dismutase | 1.73 | 1.36 |
| AOLE_14370 | *nirB* | rubredoxin-NAD(+) reductase | 1.80 | 2.00 |
| AOLE_11770 | *katE* | catalase | 1.99 | 1.72 |
| AOLE_07375 | *recA* | recombinase A | 2.14 | 2.54 |
| AOLE_17390 | *katG* | catalase | 2.38 | 3.08 |
| AOLE_08120 | - | glutaredoxin-like protein | 2.58 | 1.59 |
| AOLE_07635 | - | thioredoxin | 3.61 | 1.10 |
