# Supplementary material for: Plasmid-Encoded Tetracycline Efflux Pump Protein Alters Bacterial Stress Responses and Ecological Fitness of Acinetobacter oleivorans
Source: PLoS One. 2014 Sep 17;9(9):e107716. doi: 10.1371/journal.pone.0107716 (PMC4167995; doi:10.1371/journal.pone.0107716)
Supplement: Table S2 — Outer membrane-related gene expression profiles. (DOC) [file pone.0107716.s008.doc]

**Table S2. Outer membrane-related gene expression profiles.**

| **Locus_tag** | **Gene** | **Product** | **Fold change** | |
| --- | --- | --- | --- | --- |
|  |  |  | **DR1(pAST2)/DR1** | **DR1(pAST2)-TC/DR1-TC** |
| AOLE_17960 | *ompW* | putative outer membrane protein W | −20.03 | 1.40 |
| AOLE_00735 | *lpxC* | N-acetylglucosaminedeacetylase | −13.45 | 1.50 |
| AOLE_02355 | *mltE* | lytic murein transglycosylase | −12.71 | 1.35 |
| AOLE_09165 | *wcaG* | nucleoside-diphosphate-sugar epimerase | −11.43 | 1.72 |
| AOLE_19120 | *wecE* | glutamine--scyllo-inositol transaminase | −11.40 | 2.33 |
| AOLE_00710 | *murC* | UDP-N-acetylmuramate--L-alanine ligase | −10.83 | 2.61 |
| AOLE_05440 | *rlpA* | rare lipoprotein A | −10.70 | 3.46 |
| AOLE_00745 | *nlpD* | Peptidase family M23 family protein | −10.46 | −1.58 |
| AOLE_13130 | - | glycosyl transferase family 2 family protein | −10.20 | 1.06 |
| AOLE_19135 | *wecC* | UDP-glucose/GDP-mannose dehydrogenase | −10.19 | 2.34 |
| AOLE_05445 | *mltB* | membrane-bound lytic murein transglycosylase B | −10.11 | 1.11 |
| AOLE_18845 | *alr* | alanine racemase | −10.07 | −1.74 |
| AOLE_14065 | *mltE* | soluble lytic murein transglycosylase | −9.92 | 2.85 |
| AOLE_04275 | *htrB* | putative lipid A biosynthesis lauroylacyltransferase | −8.97 | 1.65 |
| AOLE_00705 | *murG* | N-acetylglucosaminyltransferase | −8.80 | 2.05 |
| AOLE_03950 | *mltE* | transglycosylase | −8.79 | −1.07 |
| AOLE_00390 | *murI* | glutamate racemase | −8.77 | −2.10 |
| AOLE_18465 | *mltE* | transglycosylase SLT domain-containing protein | −8.62 | −1.37 |
| AOLE_06490 | - | surface antigen family protein | −8.54 | 2.17 |
| AOLE_17560 | *lnt* | apolipoprotein N-acyltransferase | −7.79 | 2.90 |
| AOLE_03300 | *mreC* | rod shape-determining protein MreC | −7.44 | 1.83 |
| AOLE_02335 | *tonB* | hypothetical protein | −7.11 | −1.38 |
| AOLE_04235 | *tolC* | outer membrane protein | −7.11 | 2.93 |
| AOLE_14240 | *ftsI* | penicillin-binding protein 2 | −7.06 | 1.00 |
| AOLE_18125 | *tolC* | channel-tunnel spanning the outer membrane | −6.84 | 2.14 |
| AOLE_00820 | *kdtA* | 3-deoxy-D-manno-2-octulosonate transferase | −6.32 | 1.80 |
| AOLE_00720 | *ftsQ* | cell division protein | −6.29 | 1.20 |
| AOLE_17530 | *murI* | glutamate racemase | −6.25 | 1.33 |
| AOLE_14395 | *mrcB* | peptidoglycantransglycosylase | −6.21 | −1.30 |
| AOLE_09010 | *ompW* | OmpW family protein | −6.12 | 1.42 |
| AOLE_19045 | *mdoB* | sulfatase | −5.99 | 2.11 |
| AOLE_14710 | *betT* | high-affinity choline transport protein | −5.97 | −1.03 |
| AOLE_03775 | *pgsA* | bifunctional poly-gamma-glutamate biosynthesis protein | −5.95 | −1.10 |
| AOLE_16735 | *rlpB* | putative minor lipoprotein | −5.95 | 1.80 |
| AOLE_01430 | - | S-adenosyl-methyltransferase MraW | −5.84 | 2.61 |
| AOLE_03305 | *mreD* | rod shape-determining protein | −5.84 | 1.41 |
| AOLE_13005 | *tolC* | putative outer membrane efflux protein, type Isecretion protein | −5.73 | −1.37 |
| AOLE_00270 | *nlpB* | hypothetical protein | −5.71 | −1.13 |
| AOLE_10450 | *lpxB* | lipid-A-disaccharide synthase | −5.65 | 3.04 |
| AOLE_01470 | *mrcA* | putative penicillin binding protein (PonA) | −5.58 | 4.41 |
| AOLE_07005 | *murB* | UDP-N-acetylenolpyruvoylglucosamine reductase | −5.37 | 1.15 |
| AOLE_00415 | *glmU* | N-acetylglucosamine-1-phosphateuridyltransferase | −5.34 | 3.81 |
| AOLE_02710 | - | nucleoside-diphosphate-sugar epimerase | −5.29 | 1.76 |
| AOLE_13120 | *mscS* | Mechanosensitive ion channel family protein | −5.11 | 2.04 |
| AOLE_02725 | *wcaA* | putative lipopolysaccharide core biosynthesisglycosyl transferase LpsC | −4.92 | 4.07 |
| AOLE_01440 | *ftsI* | Penicillin binding protein transpeptidase domainprotein | −4.88 | 1.61 |
| AOLE_19185 | *plcC* | phospholipase C precursor | −4.73 | 1.23 |
| AOLE_10890 | *gidB* | 16S rRNA methyltransferase GidB | −4.63 | 1.96 |
| AOLE_16950 | *rfaG* | glycosyltransferase | −4.57 | −1.92 |
| AOLE_18240 | *prc* | C-terminal-processing protease | −4.42 | 2.51 |
| AOLE_18415 | *acm* | 1,4-beta-N-acetylmuramidase | −4.41 | 2.12 |
| AOLE_13800 | - | glucarate dehydratase | −4.22 | 1.20 |
| AOLE_02020 | *wcaG* | nucleoside-diphosphate-sugar epimerase | −4.11 | 3.39 |
| AOLE_16955 | *prc* | tail-specific protease | −4.03 | 3.00 |
| AOLE_07230 | *galE* | UDP-glucose 4-epimerase | −3.96 | 3.23 |
| AOLE_03135 | *acrA* | membrane-fusion protein | −3.82 | 2.25 |
| AOLE_04550 | *lepA* | GTP-binding protein LepA | −3.79 | 1.57 |
| AOLE_08735 | *oprB* | porin | −3.78 | 2.52 |
| AOLE_13135 | *cfa* | putative methyltransferase | −3.73 | 2.79 |
| AOLE_02730 | *rfaG* | glycosyltransferase | −3.71 | 2.93 |
| AOLE_00715 | *ddlA* | D-alanine-D-alanine ligase | −3.52 | 2.24 |
| AOLE_18220 | *dacC* | D-alanyl-D-alanine endopeptidase | −3.52 | 1.19 |
| AOLE_13465 | *ompA* | OmpA family protein | −3.47 | −2.56 |
| AOLE_05395 | *ompW* | outer membrane protein W | −3.43 | −1.40 |
| AOLE_15440 | *betT* | putative transporter | −3.24 | −1.58 |
| AOLE_17270 | *htrB* | lipid A biosynthesis lauroyl acyltransferase | −3.20 | 1.30 |
| AOLE_06960 | *plcC* | phospholipase C | −3.18 | 1.83 |
| AOLE_13205 | *htrB* | lauroyl/myristoyl acyltransferase | −3.07 | 16.20 |
| AOLE_09305 | *tolC* | outer membrane protein tolC | −3.07 | 2.06 |
| AOLE_10905 | *imp* | organic solvent tolerance protein OstA | −2.97 | 2.92 |
| AOLE_06085 | *racX* | aspartate racemase | −2.84 | 2.21 |
| AOLE_02520 | *lrgB* | LrgB-like family protein | −2.81 | 1.78 |
| AOLE_02980 | *oprB* | Outer membrane protein D1 (Glucose porin) | −2.80 | −1.09 |
| AOLE_04745 | *dacC* | D-alanyl-D-alanine carboxypeptidase | −2.76 | 3.18 |
| AOLE_06355 | - | Glycosyltransferase | −2.75 | −1.12 |
| AOLE_11590 | *lrgB* | putative effector of murein hydrolase | −2.67 | 1.77 |
| AOLE_14705 | *betT* | choline transport protein BetT | −2.65 | −1.18 |
| AOLE_01555 | *rfaL* | Lipid A core--O-antigen ligase | −2.62 | 2.09 |
| AOLE_00410 | *glmS* | glucosamine--fructose-6-phosphateaminotransferase | −2.55 | 3.38 |
| AOLE_12590 | *oprB* | carbohydrate porin | −2.43 | 1.75 |
| AOLE_05885 | *tolC* | RND efflux system, outer membrane lipoprotein,NodT | −2.33 | 1.52 |
| AOLE_19140 | *wza* | putative outer membrane protein | −2.32 | 1.66 |
| AOLE_05610 | *mrcB* | penicillin-binding protein 1B | −2.29 | 2.67 |
| AOLE_04995 | *nlpD* | lipoprotein | −2.29 | 1.75 |
| AOLE_19400 | *murC* | UDP-N-acetylmuramate:L-alanyl-gamma-D-glutamyl-meso-diaminopimelate ligase | −2.27 | 1.53 |
| AOLE_16345 | *vacJ* | Lipoprotein vacJ | −2.16 | 3.32 |
| AOLE_16325 | *murA* | UDP-N-acetylglucosamine1-carboxyvinyltransferase | −2.14 | 1.56 |
| AOLE_00830 | *mscS* | small-conductance mechanosensitive channel | −2.14 | 2.50 |
| AOLE_04975 | *dacC* | D-ala-D-ala-carboxypeptidase | −2.14 | 8.96 |
| AOLE_01365 | *tolC* | outer membrane protein | −2.14 | 1.84 |
| AOLE_13030 | *gutQ* | arabinose 5-phosphate isomerase | −2.11 | 1.98 |
| AOLE_00895 | *betT* | high-affinity choline transport protein | −2.09 | −1.51 |
| AOLE_04065 | *acrA* | acriflavin resistance protein A | −2.09 | 1.43 |
| AOLE_06850 | *ompW* | OmpW family protein | −2.05 | 2.19 |
| AOLE_14295 | *cutF* | Lipoprotein nlpE | −2.01 | 2.69 |
| AOLE_19070 | *galU* | UTP-glucose-1-phosphate uridylyltransferase | −1.97 | 2.05 |
| AOLE_01020 | *cfa* | putative methyltransferase | −1.96 | 3.42 |
| AOLE_19380 | *mltB* | lytic murein transglycosylase family protein | −1.95 | −1.55 |
| AOLE_19065 | *ugd* | putative UDP-glucose 6-dehydrogenase | −1.90 | 1.42 |
| AOLE_14660 | - | N-glycosyltransferase | −1.89 | −1.39 |
| AOLE_12220 | *ompA* | OmpA/MotB domain-containing protein | 1.88 | −1.62 |
| AOLE_04350 | *ompA* | peptidoglycan-associated lipoprotein | 2.00 | −1.53 |
| AOLE_09455 | *acrA* | membrane fusion protein | 2.05 | 3.02 |
| AOLE_03965 | - | lytic transglycosylase | 2.42 | −1.61 |
| AOLE_00140 | *acrA* | putative RND efflux membrane fusion protein | 2.54 | 1.61 |
| AOLE_04640 | *blc* | outer membrane lipoprotein blc | 3.05 | 4.16 |
| AOLE_09465 | *tolC* | outer membrane protein | 3.17 | 2.95 |
| AOLE_16840 | *lolA* | hypothetical protein | 3.60 | 2.11 |
| AOLE_10855 | *kdsB* | 3-deoxy-manno-octulosonate cytidylyltransferase | 3.79 | 3.49 |
| AOLE_14775 | *tonB* | TonB family protein | 4.49 | 1.20 |
| AOLE_06060 | *cfa* | cyclopropane-fatty-acyl-phospholipid synthase | 8.89 | 1.61 |
| AOLE_06030 | *blc* | outer membrane lipoprotein blc | 10.00 | 2.56 |
