# Supplementary material for: Plasmid-Encoded Tetracycline Efflux Pump Protein Alters Bacterial Stress Responses and Ecological Fitness of Acinetobacter oleivorans
Source: PLoS One. 2014 Sep 17;9(9):e107716. doi: 10.1371/journal.pone.0107716 (PMC4167995; doi:10.1371/journal.pone.0107716)
Supplement: Table S3 — Bacterial strains, plasmids, and primers used in this study. (DOC) [file pone.0107716.s009.doc]

**Table S3. Bacterial strains, plasmids, and primers used in this study.**

| **Bacterial strains/plasmids/primers** | **Description** | **Reference** |
| --- | --- | --- |
| **Strains** | | |
| *E. coli* Top10 | F-ara D 139△(ara, leu) 7697 △lacX74 galU galK rpsL (StrR) deoR ø80dlacZ△M15 endA1 nupG recA1 mcrA △(mrr–hsdRMS mcrBC) | Invitrogen |
| *E. coli* S17-1λ*pir* | Tra^+^ R6K strain, used for transformation of pCVD442 constructs | Simon et al. |
| *A. oleivorans* DR1 | Wild-type, non-naphthalene degrader, diesel oil degrader | This study |
| *A. oleivorans* DR1 (pAST2) | Strain DR1 harboring tetracycline resistance plasmid, pAST2 from activated sludge | Hong et al. |
| *A. oleivorans* DR1 (pAST2∆P*_tetH_*) | Insertion of pCVD442-*tetHtetR*::*km* in *A. oleivorans* DR1 (pAST2) | This study |
| **Plasmids** | | |
| pAST2 | Tetracycline resistance plasmid isolated from activated sludge | Hong et al. |
| pUC4K | Kanamycin resistance gene of Tn*903* in pUC4 | Taylor and Rose |
| pCVD442 | R6K *ori*, *mobRP4*, *bla*, *sacB* | Philippe et al. |
| pBBR1MCS4 | Ampicillin resistance gene, Broad-host-range vector | Kovach et al. |
| pBBR1MCS4-*tetH* | Insertion of *tetH* gene in pBBR1MCS4 | This study |
| pBBR1MCS4-*tetHtetR* | Insertion of *tetR* gene in pBBR1MCS4-*tetH* | This study |
| pBBR1MCS4-*tetHtetR*::*km* | Insertion of *km* cassette in the intergenic region between *tetH* and *tetR* in pBBR1MCS4-*tetHtetR* | This study |
| pCVD442-*tetHtetR*::*km* | Insertion of 2.2-kb fragment containing the *km* cassette in the intergenic region between *tetH* and *tetR* in pCVD442 | This study |
| **Primers** | | |
| *tetH*-F | CGC GGT ACC TTT GTG CCA ATT TCC CAG CG | This study |
| *tetH*-R | CGC GGA TCC ATG AGT GAT GTG ACT CCC GC | This study |
| *tetR*-F | CGC GGA TCC TTA ACA ACG CGT AAG CTG GC | This study |
| *tetR*-R | CGC GAG CTC AAT GGC GGA TAG GCA ACA GT | This study |
| 16s rRNA-341F | CCT ACG GGA GGC AGC AG | Watanabe et al. |
| 16s rRNA-534R | ATT ACC GCG GCT GCT GGC A | Watanabe et al. |
| AOLE_01575-F | CGGTGCATTTTCACCAAGCA | This study |
| AOLE_01575-R | CATATCCGTCGTCCGCTACC | This study |
| AOLE_04975-F | AAGCAGGTGCAAGCTTTTCG | This study |
| AOLE_04975-R | GCTGCACTCCTCCTCTTACC | This study |
| AOLE_06030-F | GCACCTTTGCCGACACAAAT | This study |
| AOLE_06030-R | CAACGGTTTTGTCCGCGTTA | This study |
| AOLE_09285-F | GCTTTTCACCAGCTTCAGGC | This study |
| AOLE_09285-R | TTAGGTCAACGGTGGCACTC | This study |
| AOLE_09780-F | GGCCTTGGTGTGTATGGTGA | This study |
| AOLE_09780-R | CGCTTCAGTGGAAGTACGGT | This study |
| AOLE_09790-F | ATTTGCCCAGCTTTCGCTTG | This study |
| AOLE_09790-R | TGGATGTGGTGTGACGACTG | This study |
| AOLE_15160-F | ATGCCCGCATTAAGGTCACA | This study |
| AOLE_15160-R | GAACCTTTCCTACACCGCCA | This study |
| AOLE_16340-F | GAAGAAGCCTCTCCACCAGC | This study |
| AOLE_16340-R | GGAAATCGGTGCAGAAGCAA | This study |
| AOLE_16925-F | ACATTTGTGTAGCAGCCGGA | This study |
| AOLE_16925-R | ACGCTATCAGGCGTTGGAAA | This study |
| AOLE_17390-F | TTAGGTAAAACCCACGGGGC | This study |
| AOLE_17390-R | GCTCCATTGTGTTGGCGTTT | This study |

Hong H, Ko HJ, Choi IG, Park W (2014) Previously undescribed plasmids recovered from activated sludge confer tetracycline resistance and phenotypic changes to *Acinetobacter oleivorans* DR1. Microb Ecol 67: 369-379.

Kovach ME, Phillips RW, Elzer PH, Roop RM 2nd, Peterson KM (1994) pBBR1MCS: a broad-host-range cloning vector. Biotechniques 16: 800-802.

Philippe N, Alcaraz JP, Coursange E, Geiselmann J, Schneider D (2004) Improvement of pCVD442, a suicide plasmid for gene allele exchange in bacteria. Plasmid 51: 246-255.

Simon R, Preifer U, Puhler A (1983) A broad host range mobilisation system for in vivo genetic engineering: transposon mutagenesis in Gram-negative bacteria. Nat Biotech 1: 784-791.

Taylor LA, Rose RE (1988) A correction in the nucleotide sequence of the Tn903 kanamycin resistance determinant in pUC4K. Nucleic Acids Res 16: 358.

Watanabe K, Kodama Y, Harayama S (2001) Design and evaluation of PCR primers to amplify bacterial 16S ribosomal DNA fragments used for community fingerprinting. J Microbiol Methods 44: 253e262.
